# The interaction between UBR7 and PRMT5 drives PDAC resistance to gemcitabine by regulating glycolysis and immune microenvironment

**DOI:** 10.1038/s41419-024-07145-z

**Published:** 2024-10-18

**Authors:** Maoxiao Feng, Qinlian Jiao, Yidan Ren, Xiaoyan Liu, Zihan Gao, Zhengjun Li, Yunshan Wang, Miaoqing Zhao, Lei Bi

**Affiliations:** 1 Department of Clinical Laboratory, Shandong Provincial Hospital Affiliated to Shandong First Medical University, Jinan, Shandong 250021 China; 2 School of Chinese Medicine, Nanjing University of Chinese Medicine, 138 Xianlin Road, Nanjing, Jiangsu 210023 China; 3 Department of pathology, Shandong Cancer Hospital and Institute, Shandong First Medical University and Shandong Academy of Medical Sciences, Jinan, Shandong 250117 China

**Keywords:** Outcomes research, Cancer therapy

## Abstract

Pancreatic ductal adenocarcinoma (PDAC) is a common malignant tumor of the digestive tract. Although gemcitabine and other therapeutic agents are effective in patients with advanced and metastatic pancreatic cancer, drug resistance has severely limited their use. However, the mechanisms of gemcitabine resistance in pancreatic cancer are poorly understood. In this study, ATAC-seq, ChIP-seq, and RNA-seq were performed to compare chromatin accessibility and gene expression in a patient-derived tumor xenograft (PDX) model of pancreatic cancer with or without gemcitabine resistance. Analyzing these sequencing data, we found a dramatic change in chromatin accessibility in the PDX model of gemcitabine-resistant tissues and identified a key gene, UBR7, which plays an important role in mediating gemcitabine resistance. Further research found that depletion of UBR7 significantly increased pancreatic carcinogenesis and the immunosuppressive microenvironment. Mechanistically, depleted UBR7 increased the stability of PRMT5, thereby promoting glycolysis in pancreatic cancer cells. Finally, an inhibitor that blocks PRMT5 (DS-437) significantly reduced gemcitabine resistance in pancreatic cancer caused by UBR7 depletion. In conclusion, our results illustrate that the UBR7-PRMT5 axis is a key metabolic regulator of PDAC and a promising target for the clinical treatment of gemcitabine resistance in PDAC.

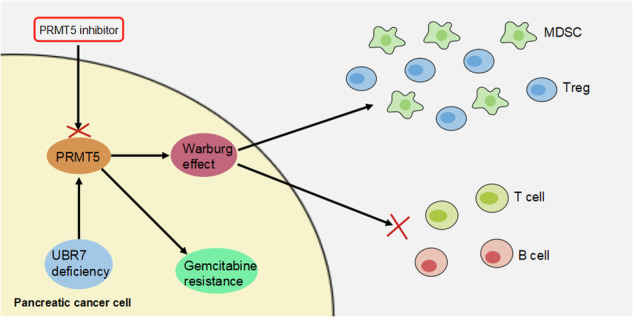

## Introduction

The incidence of PDAC is increasing worldwide. Despite the current best treatment methods, the mortality rate is still >90%, which highlights the urgent need to find effective treatments [1]. The current long-term relief from standard therapies such as chemotherapy, surgery, and targeted therapy is still scarce, which emphasizes the need for new methods [2]. The use of gemcitabine is still the first-line standard treatment for pancreatic cancer [3]. However, with the use of chemotherapy drugs, pancreatic cancer patients often experience drug resistance and insensitivity, which leads to extremely poor patient prognosis and survival [4–6]. Studies have shown that multiple mechanisms are involved in regulating the resistance of pancreatic cancer to gemcitabine, including noncoding RNA [7], transcription factors [8], and the pancreatic cancer microenvironment [9–11].

In addition to the acquisition of resistance-conferring genetic mutations, increasing evidence suggests that epigenetic mechanisms are also closely involved in this process [12]. H3K27ac is a histone acetylation modification indicative of active chromatin regions [13, 14]. Significant alterations in histone H3 lysine 27 acetylation (H3K27ac) were found in anaplastic lymphomas resistant to anaplastic lymphoma kinase (ALK) inhibitors, thereby remodeling the expression levels of resistance-induced miRNAs and mRNAs [15]. In addition, lysine demethylase 5 A (KDM5A) increases resistance to multiple anticancer drugs, including breast cancer resistance to erlotinib and lung cancer cell resistance to gefitinib, by removing histone methylation [16, 17]. However, much remains unknown about the epigenetic regulation of systems in gemcitabine-resistant PDAC, such as epigenetic plasticity and transcriptional regulation.

In this study, we constructed gemcitabine-resistant and gemcitabine-sensitive PDAC-PDX mouse models and explored their epigenetic and chromosomal accessibility. Compared with the PDAC-PDX tissues of the sensitive group, we found that the level of H3K27ac was significantly reduced in gemcitabine-resistant PDAC-PDX tissues. Combined with transcriptomic analysis, we found that the expression level of UBR7 was significantly reduced in gemcitabine-resistant PDAC-PDX tissues and could also mediate the occurrence and progression of PDAC.

Mechanistically, we showed that depleting UBR7 increased PRMT5 protein stability. Studies have also shown that PRMT5 promotes Warburg anaerobic glycolysis in human breast and pancreatic cancer cells [18, 19], and PRMT5 knockdown also reduces glucose consumption or the extracellular acidification rate (ECAR) [20]. In addition, knockdown of PRMT5 in human transformed human Jurkat T cells resulted in reduced glycolytic metabolism, as measured by ECAR, and reduced oxidative phosphorylation, as measured by the oxygen consumption rate (OCR) [21]. Therefore, we speculated that UBR7 regulated glycolysis in pancreatic cancer by regulating the expression of PRMT5, thereby promoting drug resistance. Furthermore, we also found that depletion of UBR7 increased immunosuppressive cells (Tregs and myeloid-derived suppressor cells) in the PDAC microenvironment. Finally, PRMT5 inhibitors increased the sensitivity of UBR7-depleted PDACs to gemcitabine.

## Materials and methods

### Cells

Pancreatic cancer cell lines (PANC-1, BxPC-3, S2-013, ASPC-1, pan02 and MIAPACA-2), pancreatic ductal cell line (HPNE) and mouse monocyte macrophage RAW264.7 were from the Chinese Academy of Science Cell Bank. ASPC-1 and pan02 cells were cultured in RPMI 1640 medium (Gibco, USA) containing 10% fetal bovine serum (FBS; Gibco, USA), 100 IU/mL penicillin, and 100 μg/mL streptomycin (Gibco, USA). The remaining cells were cultured in DMEM medium containing 10% FBS, 100 IU/mL penicillin, and 100 μg/mL streptomycin. All cell lines were maintained in a 5% CO_2_ incubator at 37 °C.

### Antibodies and chemicals

In this study, we used the following antibodies: anti-UBR7 (Abcam Cat# ab228911), anti-PRMT5 (Abcam Cat# ab109451), anti-Myc (Cell Signaling Technology# 9B11), anti-HA (Cell Signaling Technology# 3724), anti-Flag (Cell Signaling Technology# 14793), anti-UB (Abcam Cat# ab272168), anti-MDR1 (Cell Signaling Technology# 13342S), anti-LRP (Affinity # DF2935), RRM1(Cell Signaling Technology# 3388) and Anti-β-Actin (Abcam Cat# ab8227). The following chemicals were used: gemcitabine (T0251, Topscience), MG132 (HY-13259, MedChemExpress) and Cycloheximide (HY-12320, MedChemExpress) and DS-437 (HY-124131, MedChemExpress).

### In vitro ubiquitination assays

A ubiquitination kit (biotechne R&D SYSTEMS, K-980B) was used to perform ubiquitination analysis according to the manufacturer’s recommended protocol.

### PDAC tissue and gemcitabine-resistant or gemcitabine-sensitive PDX models

We purchased 4-week-old male NSG mice from Shanghai Model Organisms Center Inc. (Shanghai, China). Tumor tissue from PDAC patients was obtained with the written consent of all patients. PDAC tissue from patients was cut into 3 × 3 mm blocks and transplanted subcutaneously into NSG mice. PDX tumors were then maintained by serial implantation into NSG mice. Gemcitabine treatment was used to induce drug-resistant PDX in PDX model mice. The standard feature of the drug-resistant PDX model is the tumor growth rate. For related data, please refer to Supplementary Fig. 1A, B. The acquisition of tumor tissue from PDAC patients and the PDX model mice was followed the guidelines of the Ethics Committee of the Second Hospital of Shandong University. The investigator was blinded to the group allocation of the mice during the experiment. The sample size is described in the corresponding figure legend. No animals were excluded from the analysis.

### Primary isolation and culture of pancreatic cancer tissue

Pancreatic tumor tissues from PDX model mice were taken under aseptic conditions, immediately washed with D-hanks under an ultra-clean bench to remove fat, blood clots and necrotic tissues, and cut into pieces of 1 mm in diameter and placed in 1.5% collagenase. After digestion, filter through a 160-mesh strainer to remove large tissue clumps. The cells are collected by centrifugation. The cells were transferred to culture flask 1 in RPMI 1640 medium with 10% FBS. After half an hour, the supernatant was transferred to culture flask 2, and after another half an hour, the supernatant was transferred to culture flask 3. The cells in culture flask 3 were considered as primary pancreatic tumor cells. EGF, transferrin, insulin and hydrocortisone were added to the culture medium to promote cell growth and proliferation.

### Mice

*Ubr7*^*-/-*^ mice were purchased from Cyagen Biosciences Inc. (Guangzhou, China). *K-ras*^*LSL. G12D*^ and *Pdx1-Cre* mice were purchased from the Jackson Laboratory (Bar Harbor, ME). KC mice were obtained by crossing *K-ras*^*LSL. G12D*^ and *Pdx1-Cre* mice, which expressed Kras mutations and developed into endogenous pancreatic cancer [22]. *Ubr7*^*–/–*^ mice were crossed with KC mice to generate KC; *Ubr7*^*–/–*^ mice. The procedure for PDX model construction was as follows: Fresh tumor tissue samples were collected from patients with pancreatic cancer, quickly stored in organ preservation solution, and transferred to a biological safety cabinet within 1 h. The samples were then cut into small tumor pieces of approximately 0.2 cm and transplanted into immunodeficient NSG mice. All animal procedures were approved by the Second Hospital of Shandong University Institutional Animal Care and Use Committee. Human tissues were obtained under an institutional review board-approved protocol.

### Subcutaneous xenograft tumor model

We purchased 4-week-old male BALB/c nude mice from Shanghai Model Organisms Center Inc. (Shanghai, China). After 1 week of adaptive feeding, the mice were randomly divideo into three groups (*n* = 5 each). 3 × 10^6^ stable PANC-1-GEM cells (Ctrl, UBR7 or UBR7 + PRMT5) were resuspended in 100 μL of sterile PBS and injected subcutaneously into the armpit of nude mice. After 4 weeks, the nude mice in each group were killed by cervical dislocation, and the transplanted tumors of the nude mice were removed and weighed. The investigator was blinded to the group allocation of the mice during the experiment. No animals were excluded from the analysis.

### ChIP-seq

Collagenase IV (17104019, Gibco, 4 mg/mL) / dispase II (17105041, Gibco, 4 mg/mL)/RPMI was used to lyse tumor tissues to obtain cell lysates. These cells were fixed with formaldehyde, snap-frozen in liquid nitrogen and lysed to obtain DNA fragments. Libraries were quantified using a Bioanalyzer 2100 (Agilent) and sequenced on an Illumina HiSeq 2000 Sequencer. Sequencing tags were mapped to the Homo sapiens (human) reference genome (hg19) using Bowtie 267. MACS (2.1.0) uses uniquely mapped labels for peak calling to identify ChIP-Seq-enriched regions in the background. A q-value enrichment threshold of 5 × 10^–2^ was used for all datasets. Normalized genome-wide signal coverage trajectories from raw read alignment files were constructed by MACS2, UCSC tools (http://hgdownload.cse.ucsc.edu/admin/exe/linux.x86_64/bedGraphToBigWig/bedClip) and BEDTools (RRID:SCR_006646, http:///code.google.com/p/bedtools/). Visualization of ChIP-seq signals in enriched genomic regions (avgprof and heatmaps) was achieved by using ngs.plot (https://github.com/shenlab-sinai/ngsplot).

### Assay for transposase-accessible chromatin using sequencing (ATAC-seq)

Collagenase IV (17104019, Gibco, 4 mg/mL)/dispase II (17105041, Gibco, 4 mg/mL)/RPMI was used to lyse tumor tissues to obtain cell lysates. The cells were pelleted and resuspended in 25 μl lysis buffer and then pelleted again. The nuclear pellet was resuspended in 25 μl of transposition reaction mixture containing Tn5 transposase from the Nextera DNA Sample Prep Kit (Illumina) and incubated at 37 °C for 30 min. Then, the MinElute Purification Kit (Qiagen) was used to purify the transposase-related DNA. To amplify the library, the KAPA Real-Time Library Amplification Kit (KAPA Biosystems) with Nextera indexed primers was used to amplify the DNA for 12 cycles. Total amplified DNA was purified using AmPureXP beads. TapeStation checks the amount and size of amplified DNA to confirm that independent samples show similar fragment distributions. HiSeq 4000 and paired-end sequencing (Illumina) were used to sequence the library. Repeats were generated from three independent experiments.

### RNA sequencing

For mRNA sequencing (RNA-seq) analysis of tumor tissues, freshly dissected tumor samples were frozen in RNase-free tubes containing liquid nitrogen and stored at −80 °C. A Direct-zol RNA Kit (Zymo Research) was used to extract total RNA. The RNA 6000 Nano Kit was used for quality control analysis on the Bioanalyzer 2100 (Agilent). Through the core facilities of MDACC Sequencing and ncRNA Program, the Illumina TruSeq chained mRNAseq library and high-output sequencing PE 75 × 75 nt were used for total mRNA sequencing on NextSeq 500 (Illumina). The raw sequencing data from the Illumina platform were converted into Fastq files and aligned with the reference genome mm10 using the Spliced Transcripts Alignment to a Reference (STAR) algorithm. The HTSeq counts were then used to generate raw counts for each gene. Then, the raw counts were analyzed by DESeq (RRID:SCR_000154), and data processing, standardization and differential expression analysis were performed according to standard procedures. The unsupervised hierarchical clustering heatmap is based on differentially expressed genes between sensitive and resistant tumors (defined by DESeq P value < 0.05 and fold change> 1.5). The color scheme of the heatmap represents the Row Z score distribution. Plot of the heatmap of the differentially expressed genes with the log2-fold change of the specified gene. Gene set enrichment analysis (GSEA; RRID:SCR_003199, Broad Institute) was used to perform functional classification and pathway reconstruction of RNA-seq data.

### Single-cell RNA-sequencing (scRNA-seq)

Fresh mouse pancreatic tumor tissue was minced with a sterilized lancet, and collagenase IV (17104019, Gibco, 4 mg/mL)/dispase II (17105041, Gibco, 4 mg/mL)/RPMI was used at 37 °C for 0.5 h. The cells were filtered through a 70 mm filter and resuspended in PBS/2% FBS. The single-cell suspension was stained with the live/dead viability dye eFluor 780 (65-0865-14, eBioscience), filtered through a 40 mm sieve, and then analyzed for each cell using an Aria II sorter (BD Biosciences). CD45^+^ magnetic beads were used to sort lymphocytes from living cells. The median gene read per cell was approximately 2300. The percentage of total mapped sequence reads with a QC30 threshold in the RNA read score was higher than 70%. The cell fraction reading was approximately 80%. Single-cell gel beads were prepared in emulsion (GEM) generation and barcode, GEM-RT postcleanup and cDNA amplification, library construction, and Illumina Ready Sequencing library generation were prepared in accordance with the manufacturer’s guidelines. The high-sensitivity dsDNA Qubit kit was used to estimate cDNA and library concentrations. An HS DNA Bioanalyzer was used to quantify cDNA. The DNA 1000 Bioanalyzer was used to quantify the library. The “c-loupe” file was generated using the Cell Ranger software pipeline in accordance with the manufacturer’s guidelines. From ungraded KCs or KCs, UBR7^–/–^ tumor cells were analyzed using the 10X Genomics Chromium controller and Single Cell 3’ Reagent Kits v2 package. After capture and lysis, cDNA was synthesized and amplified to construct an Illumina sequencing library. A library of approximately 1000 cells per sample was sequenced using an Illumina Nextseq 500. The library Seurat version 3.5.3, dplyr and cowplot were loaded into R to explore QC indicators, filter cells, normalize data, cluster cells, and identify cluster biomarkers. To filter out low-quality cells, thresholds of at least 200 and at most 7000 genes per cell were used. Cells with more than 10% of the mitochondrial genome were also removed for further analysis. The “RunUMAP” function was used to cluster cells. Based on the “JackStrawPlot” and “Elbow-Plot” functions, the first 27 principal components were used for UMAP projection and cluster analysis. The “FindAllMarkers” function was used to identify the specific markers of each cell cluster. The “DoHeatmap” function was used to display the top 10 genes in each cluster.

### qRT–PCR

A Direct-zol RNA kit (Zymo Research) was used to extract total RNA from cells, a reverse transcription kit (Applied Biosystems) was used to process cDNA synthesis, and SYBR Green Master Mix (Applied Biosystems) was used for qRT–PCR. The expression level of the designated gene was normalized to GAPDH.

### Flow cytometry

Spleen tissue or pancreatic cancer tissue was placed in cold RPMI 1640 medium containing 1 mg/ml collagenase IV (Worthington Biochemical Company) and 2 U/ml DNase I (Promega) and cut into submillimeter pieces with scissors. Then, the tissue was incubated at 37 °C with gentle shaking every 5 min for 30 min. The sample was passed through a 70 μm sieve and centrifuged at 350 × *g* for 5 min. For flow cytometry experiments, the cell pellet was resuspended in cold PBS containing 1% FBS. After blocking FcγRIII/II with αCD16/αCD32 mAb (eBioscience), the cells were incubated with 1 μg of fluorescently coupled mAb to label mouse CD44 (IM7), CD62L (MEL-14), IL-17 (TC11-18H10. 1), CD25 (3C7), CD206 (C068C2), PD-1 (29 F.1A12), CD3 (17A2), CD4 (RM4-5), CD8 (53-6.7), CD45 (30-F11), CD11c (N418), IFN-γ (XMG1.2), TNF (MP6-XT22; all BioLegend), T-bet (eBio4B10), FoxP3 (FJK-16; all eBioscience), GSDMD (HPA044487) and IL-1β (166931; all research and development systems). A Fixation Permeabilization Solution Kit (BD) was used for intranuclear staining of cytokines and transcription factors. Flow cytometry was performed on an LSRII flow cytometer (BD), and FlowJo (RRID:SCR_008520, v.10.1; Tree Star) was used to analyze the data.

### Mass spectrometry

After HEK293T cells were transfected with Flag-UBR7 for 24 h, the anti-Flag antibody was used to enrich the proteins bound to UBR7. SDS–PAGE was performed with Coomassie Blue, and the binding protein was identified by mass spectrometry (Maxis II, Institute of Advanced Medicine, Shandong University). Mascot software was used to identify protein names, and the binding protein was required to contain more than two high-fidelity peptides (IonScore> 20).

### Immunochemistry

For paraffin-fixed samples, mouse tissues were fixed in 10% neutral buffered formalin, embedded in paraffin, and sectioned at a thickness of 5 mm. The sections were stained with hematoxylin and eosin (H&E). For immunolabeling experiments, the sections were developed by DAB and counterstained with hematoxylin. All the above staining, imaging and quantification procedures were performed without knowing the identity and phenotype of the sample.

### Induction of M2 macrophages

The original RAW264.7 cell line is an M0 macrophage. To generate M2 polarized macrophages, RAW264.7 cells were treated with IL-4 (20 ng/ml) for 24 h. To further investigate the role of the tumor microenvironment on macrophage polarization, we constructed a co-culture system of Ubr7^WT^ and Ubr7^–/–^ mouse pancreatic cell supernatants with RAW264.7 macrophages. RAW264.7 cells were pretreated with tumor cell conditioned medium for 24 h to simulate tumor-associated macrophages.

### Statistical analysis

Data are presented as the mean ± standard error. Survival was measured according to the Kaplan–Meier method. Statistical significance was determined by Student’s t test and Wilcoxon test using Prism 7 (GraphPad Prism, RRID:SCR_002798). *P* values < 0.05 were considered significant.

## Results

### Landscape of epigenetic regulation regions in sensitive and resistant PDAC-PDX mice

**Fig. 1:**
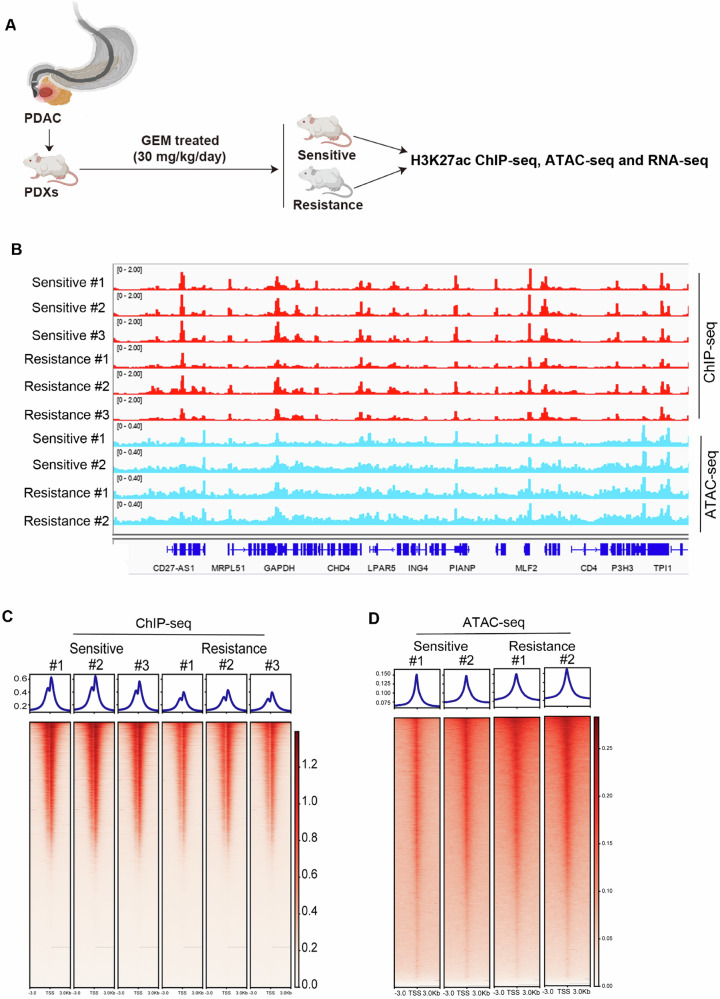
Landscape of H3K27ac and DNA accessibility in sensitive and resistant PDX samples. **A** PDAC patient tissues were collected to construct a mouse PDX model, and PDX mice were treated with GEM and subjected to ChIP-seq, ATAC-seq and RNA-seq. **B** Normalized H2K27ac chromatin immunoprecipitation sequencing profiles at a locus in sensitive and resistant PDX samples, together with normalized ATAC-seq profiles. **C** The average intensity curves (top panel) and heatmaps (bottom panel) for H3K27ac signals in genomic regions (3 kb upstream and downstream of the transcription start site (TSS)). **D** The average intensity curves (top panel) and heatmaps (bottom panel) for chromatin accessibility signals in genomic regions (3 kb upstream and downstream of the transcription start site (TSS)).

To explore the new potential mechanisms that lead to drug resistance in pancreatic cancer, we collected tumor tissues from patients with pancreatic ductal cancer to construct a PDX model and treated the mice with gemcitabine. During the treatment, we obtained PDX mice in the sensitive group (*n* = 10) and PDX mice in the resistant group (*n* = 5) (Supplementary Fig. S1A). We examined the expression of traditional resistance markers multidrug resistance 1 (MDR1), lung-resistance protein (LRP) and ribonucleotide reductase Ml (RRM1) in the tissues of both groups to confirm the correct grouping. The results showed that these markers were more highly expressed in the resistant group (Supplementary Fig. S1B). Subsequently, we collected tumor tissues for H3K27ac ChIP-seq, ATAC-seq and RNA-seq and analyzed the epigenetic landscape and differentially expressed genes (Fig. 1A). We identified 98,145 and 26,402 merged H3K27ac and ATAC-seq peaks, respectively. Visual inspection and quantitative analysis indicated that these data were of high quality with a strong signal-to-background ratio (Fig. 1B). Correlation and clustering analysis of ChIP-seq and ATAC-seq recapitulated the group classification of these samples, separating the sensitive and resistant groups (Supplementary Fig. S1C, S1D). The peaks of H3K27ac and ATAC-seq were frequent in promoters, introns, and distal intergenic regions (Supplementary Fig. S1E, S1F). Next, we explored the global profiles based on H3K27ac and ATAC-seq data. As shown, the H3K27ac signals were lower in the resistant group than in the sensitive group (Fig. 1C). This suggested that the chromosomes in the resistant group were in an inactive state, resulting in a loss of function of many genes. However, the chromatin accessibility in resistance was similar like that of the sensitive group (Fig. 1D). This may be because the resistant group is in a similar physiological state to the sensitive group, or the resistance may not be caused by changes in chromatin status. Overall, these results demonstrate the feasibility of obtaining high-quality epigenomic data from sensitive and resistant PDX mice.

### Dynamic changes of H3K27ac signaling in PDX mice

**Fig. 2:**
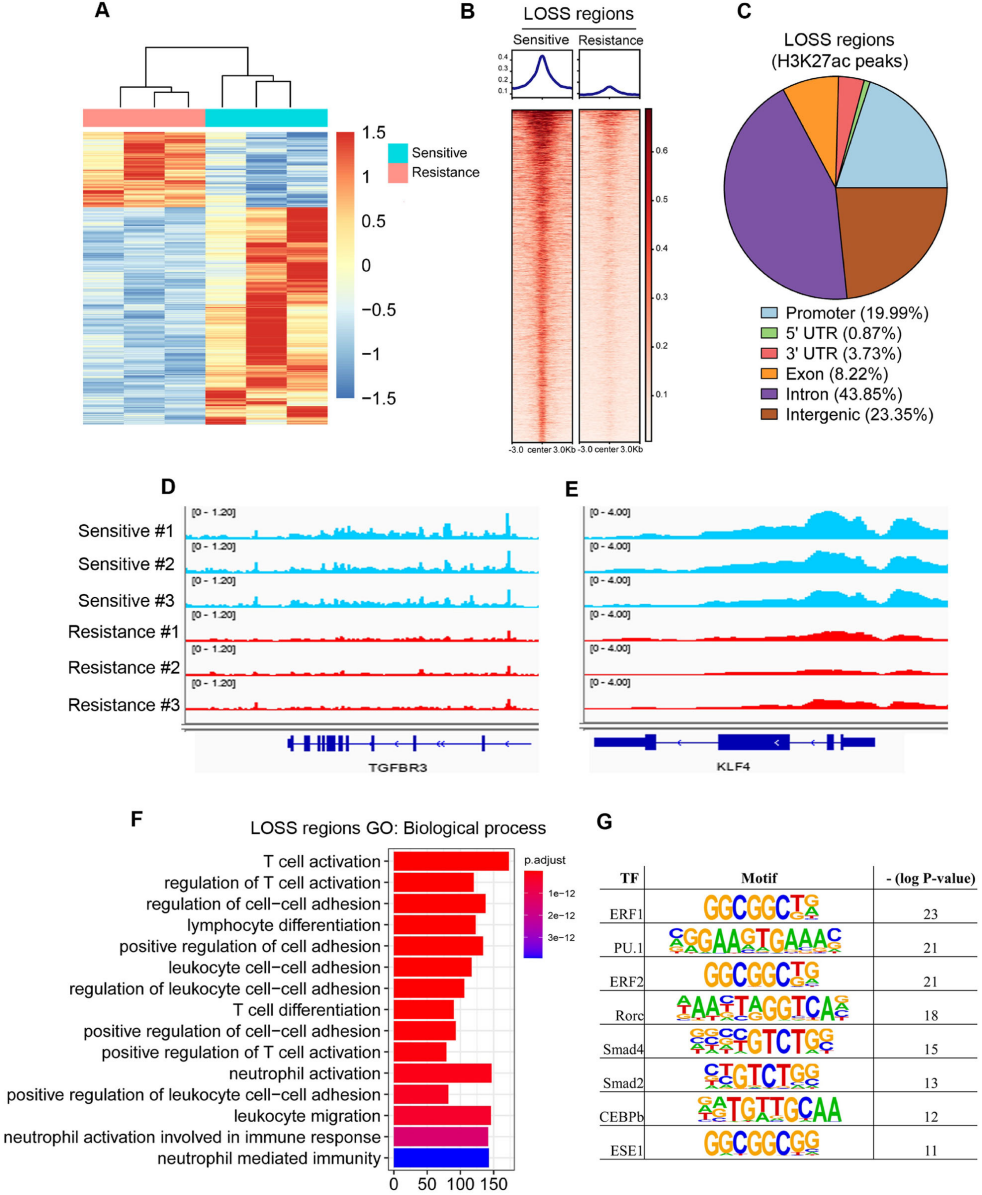
Changes in H3K27ac signals in sensitive and resistant PDX samples. **A** Heatmap analysis showed that sensitive and resistant PDX samples could be distinguished by different H3K27ac signals. **B** Heatmap representation of LOSS regions based on H3K27ac occupancy in PDX models. The 7980 LOSS regions were identified from the union of 98,145 H3K27ac peaks. Each row represents a single region (*n* = 7980). **C** Pie chart showing the genomic annotations of 7980 LOSS regions according to the location of a given peak. **D** H3K27ac signals at the TGFBR3 gene locus in GEM sensitive and resistant PDX samples. **E** H3K27ac signals at the KLF4 gene locus in GEM sensitive and resistant PDX samples. **F**, **G** GO (Gene Ontology) analysis and Motifs indicated the LOSS peak-related signaling pathway in resistant samples.

We next compared genome-wide signals of H3K27ac in sensitive and resistant PDX mice. Our analysis recovered 10,742 differential peaks (Fig. 2A), including 7980 decreased regions and 2762 increased H3K27ac regions, which we hereafter refer to as GAIN and LOSS regions, respectively (Fig. 2B and Supplementary Fig. S2A). More than 80% of GAIN and LOSS regions lied outside of promoter regions, suggesting that they represent enhancer elements (Fig. 2C and Supplementary Fig. S2B). This analysis suggests that the resistance transition in the PDX model is accompanied by dramatic changes in H3K27 acetylation. For example, the tumor suppressor-related proteins TGFBR3 [23] and KLF4 [24] both failed to open in the resistant group compared with the sensitive group (Fig. 2D, E). Gene ontology (GO) analysis for these LOSS peaks identified some immune-associated pathways, including T-cell activation, lymphocyte differentiation, leukocyte cell–cell adhesion and T-cell differentiation, indicating that the suppression of immune signaling played an important role in gemcitabine resistance in PDAC (Fig. 2F). GAIN peaks were enriched in adherens junction organization, regulation of Ras protein signal transduction and cell junction assembly pathways (Supplementary Fig. S2C). We performed motif finding using the hypergeometric optimization of motif enrichment (HOMER) algorithm to determine whether specific motifs were enriched in these differential peaks. In LOSS peaks, the top enriched motif was ERF1 (Fig. 2G). In GAIN peaks, the top enriched motif was NF1 (Supplementary Fig. S2D). Taken together, these results indicated that immunosuppression might play a vital role in gemcitabine resistance in PDAC.

### UBR7 is involved in the gemcitabine resistance of pancreatic cancer

**Fig. 3:**
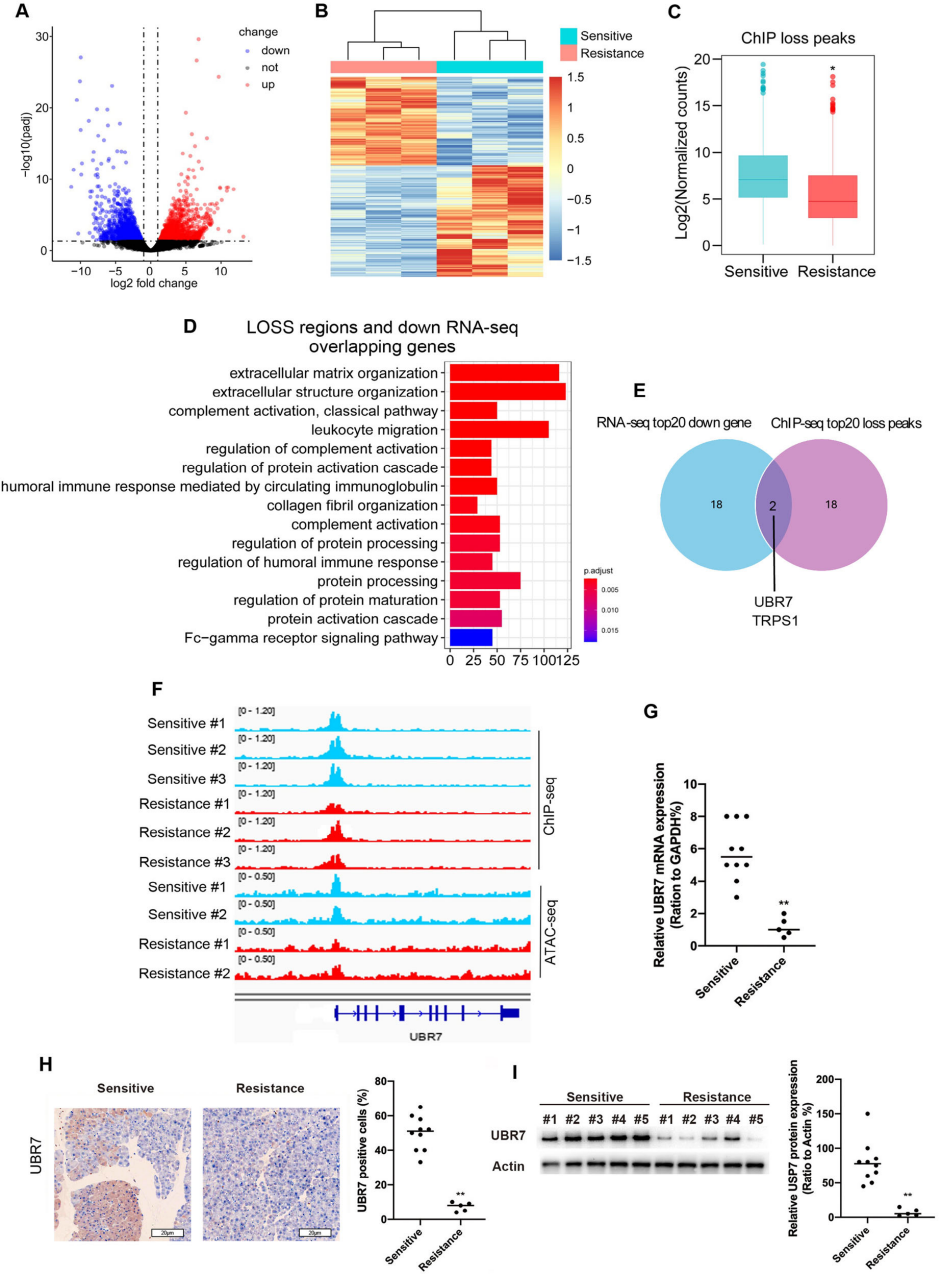
UBR7 is involved in the gemcitabine resistance of pancreatic cancer. **A** Volcano plot showing differentially expressed genes in sensitive and resistant PDAC-PDX tumor tissues. **B** Heatmap showing the differentially expressed genes identified by RNA-seq in sensitive and resistant PDAC-PDX tumor tissues. **C** Boxplots of RNA-seq counts per transcript from the sensitive and resistant tumor tissues for LOSS peak-annotated genes. **D** GO analysis was performed on the LOSS peak-associated genes that were also downregulated in the RNA-seq analysis. **E** The intersection of the top 20 decreased genes and top 20 differential LOSS peak-associated genes in resistant tumor tissues compared with sensitive tumor tissues. **F** H3K27ac and ATAC signals at the UBR7 gene locus. **G** RT-qPCR assays were performed to detect UBR7 expression in sensitive and resistant tumor tissues. **H** Representative images (left) and quantification (right) of IHC staining for UBR7 in sensitive and resistant tumor tissues. Scale bars, 20 μm. **I** Immunoblotting to measure UBR7 protein levels in sensitive and resistant tumor tissues. *p < 0.05; **p < 0.01. Error bars represent the standard error for three technical replicates.

RNA was prepared from sensitive and resistant tumor tissues. Global reprogramming of the transcriptome was detected in the resistant group compared with the sensitive group (Supplementary Fig. S3A). A total of 3420 genes were differentially expressed in the resistant group compared with the sensitive group, including 1908 upregulated genes and 1512 downregulated genes (Fig. 3A, B). We found that, on average, gene loci that had a LOSS H3K27ac signal had decreased expression (*p* < 0.05, Student’s *t* test) (Fig. 3C), indicating a high correlation of epigenetic and RNA profiling. GO analysis of the genes with both significantly downregulated RNA expression and decreased H3K27ac occupancy showed “Humoral immune response” as one of the top pathways (Fig. 3D), indicating immunosuppression in the resistant group. However, GO analysis of the genes with both upregulated gene expression and increased H3K27ac signals showed that some catabolic processes were enhanced in the resistant group, suggesting a role of metabolism in gemcitabine resistance in pancreatic cancer (Supplementary Fig. S3B). To identify genes essential for gemcitabine resistance in pancreatic cancer, we selected the top 20 LOSS peak-associated genes and the top 20 downregulated genes and then intersected them. Both UBR7 and TRPS1 were identified (Fig. 3E). We found that UBR7 lacked H3K27ac occupancy in its promoter in the resistant group compared with the sensitive group, while UBR7 showed decreased open chromatin in its promoter in the resistant group compared with the sensitive group (Fig. 3F). However, we found that TRPS1 only showed reduced H3K27ac signals but was not changed in the resistant group compared with the sensitive group (Supplementary Fig. S3C). Thus, we hypothesized that UBR7 might play an important role in gemcitabine resistance in pancreatic cancer.

We detected the expression level of UBR7 in the PDX tumor tissues of the sensitive group and the resistant group. The results showed that the protein and mRNA levels of UBR7 in the tumor tissues of the resistant group were significantly lower than those in the PDX tumor tissues of the sensitive group (Fig. 3G–I). In addition, the protein and mRNA levels of UBR7 in the gemcitabine-resistant cell line PANC-1-GEM were significantly lower than those in normal pancreatic cancer cell lines (Supplementary Fig. S4A, S4B). At the same time, we also extracted primary cells from PDX tumor tissues of the sensitive and resistant groups for culture. The expression of UBR7 was knocked down in the primary cells of the sensitive group, on the contrary, the expression of UBR7 was increased in the primary cells of the resistant group. Gemcitabine (1uM) was applied to the above cells and cell viability was detected after 48 h. The results showed that sensitive cells became more viable after knockdown of UBR7 expression, whereas resistant cells showed decreased cell viability after overexpression of UBR7 (Supplementary Fig. S4C). These results indicate that UBR7 may be involved in the gemcitabine resistance mechanism of PDAC.

### Deletion of UBR7 promotes PDAC tumorigenesis

**Fig. 4:**
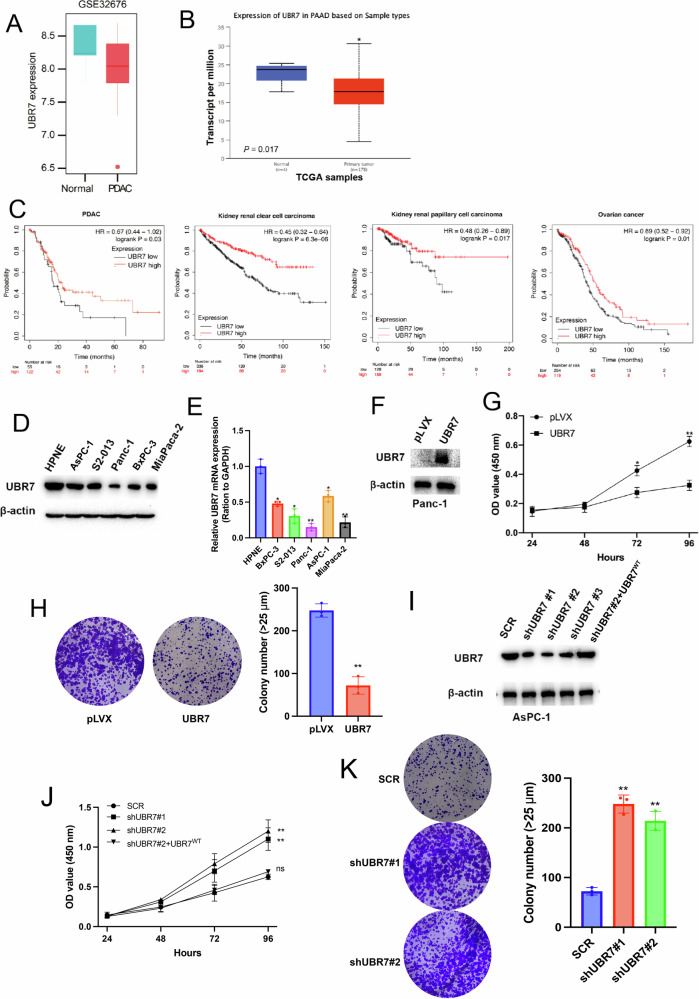
UBR7 is involved in the progression of PDAC. **A**, **B** UBR7 expression levels in normal and PDAC tissues from GEO (GSE32676) and TCGA data (Analyzed using the limma package in R software). **C** The relationship of disease-free survival of patients with PDAC and the relationship of UBR7 expression with overall survival of Kidney renal clear cell carcinoma (*p* = 6.3e–06), Kidney renal papillary cell carcinoma (*P* = 0.017) or Ovarian cancer (*P* = 0.01) (http://kmplot.com/analysis/index.php?p=service). **D**, **E** UBR7 protein expression level and mRNA expression level in pancreatic cancer cell lines and normal pancreatic cells. **F** Stable overexpression of UBR7 in Panc-1 cells. MTT and clone formation experiment detects the cell viability (**G**) and proliferation (**H**) of Panc-1 cells overexpressing UBR7. **I** Western blot detection of UBR7 knockdown and complementation levels in AsPC-1 cells. **J** MTT to detect the cell viability of AsPC-1 cells with UBR7 knockdown and UBR7 complementation. **K** Clone Formation Test to detect the cell proliferation of AsPC-1 cells with UBR7 knockdown. *p < 0.05, **p < 0.01. Data are presented as mean ± standard error.

From the above results, we found that UBR7 is involved in regulating gemcitabine resistance in PDAC. We further explored the effect of UBR7 on PDAC tumorigenesis. The analysis of UBR7 expression level and survival time in PDAC patients and normal patients showed that UBR7 was significantly reduced in PDAC (Fig. 4A, B), and low UBR7 expression was associated with poor prognosis in patients with multiple tumors (PDAC, kidney renal clear cell carcinoma, kidney renal papillary cell carcinoma and ovarian cancer) (Fig. 4C). In addition, we found that the expression level of UBR7 in normal pancreatic cells was significantly higher than that in pancreatic cancer cells (Fig. 4D, E). Overexpression of UBR7 in pancreatic cancer cells significantly reduced cell viability and proliferation capacity (Fig. 4F–H). In contrast, knocking down UBR7 significantly increased cell viability (Fig. 4I, J) and proliferation ability (Fig. K). However, when the UBR7^WT^ mutant was added back to shUBR7#2 AsPC-1 samples, the cell viability decreased significantly (Fig. 4J). These results also indicate that UBR7 is involved in the progression of pancreatic tumors.

**Fig. 5:**
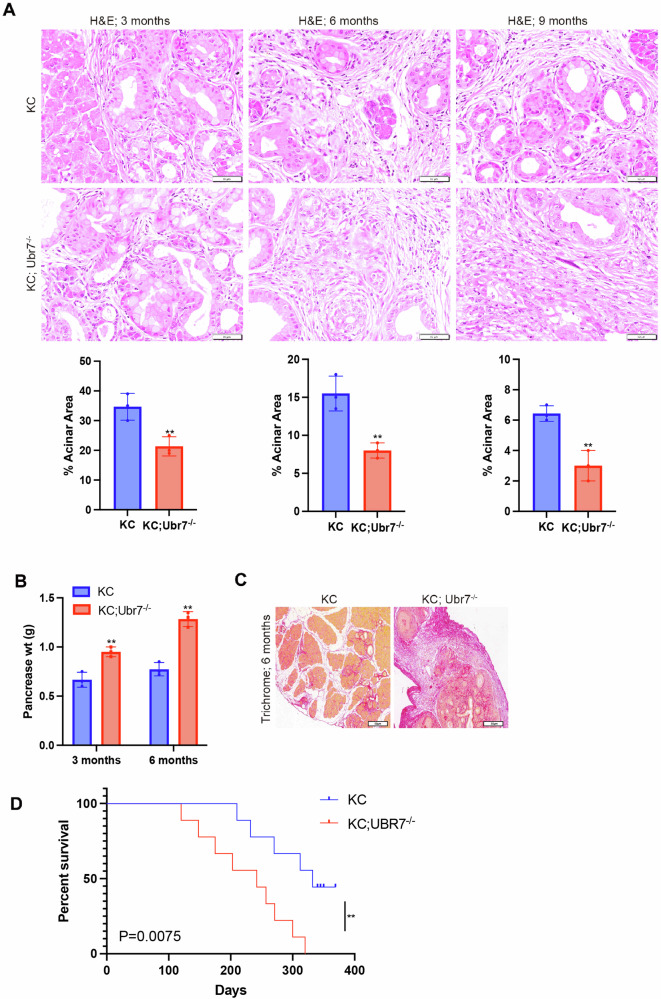
Deletion of UBR7 promotes PDAC tumorigenesis. **A** KC and KC;*Ubr7*^–/–^ mice were sacrificed at 3, 6 or 9 months of age (6–7 mice at each time point). A representative H&E-stained section is shown. The percentage of the pancreas area occupied by the intact acinar structure and the proportion showing the normal shape were calculated. **B** Comparison of pancreatic weight in KC and KC; *Ubr7*^–/–^ mice at 3 and 6 months old. **C** A representative Trichrome staining section is shown in pancreases of 6-month-old KC and KC; Ubr7^–/–^ mice. **D** Kaplan–Meier survival analysis was performed on KC (*n*) and KC; Ubr7^–/–^ (*n*) mice. **p < 0.01. Error bars represent the standard error for three technical replicates. The scale bar is 50 μm in (**A**, **C**).

We constructed a *Ubr*7^–/–^ whole-body knockout gene mouse and obtained KC;*Ubr*7^–/–^ by crossing with LSL-*Kras*^G12D^;Pdx-1-Cre (KC) mice. Compared with the KC control, the age-matched KC;*Ubr*7^–/–^ pancreas showed faster development of pancreatic dysplasia (Fig. 5A), increased pancreatic weight (Fig. 5B), increased fibrosis around the pancreas (Fig. 5C) and shortened lifespan (Fig. 5D). These data indicate that depletion of UBR7 promotes the accelerated progression of pancreatic tumors.

### UBR7 degrades PRMT5 through K48 ubiquitination

**Fig. 6:**
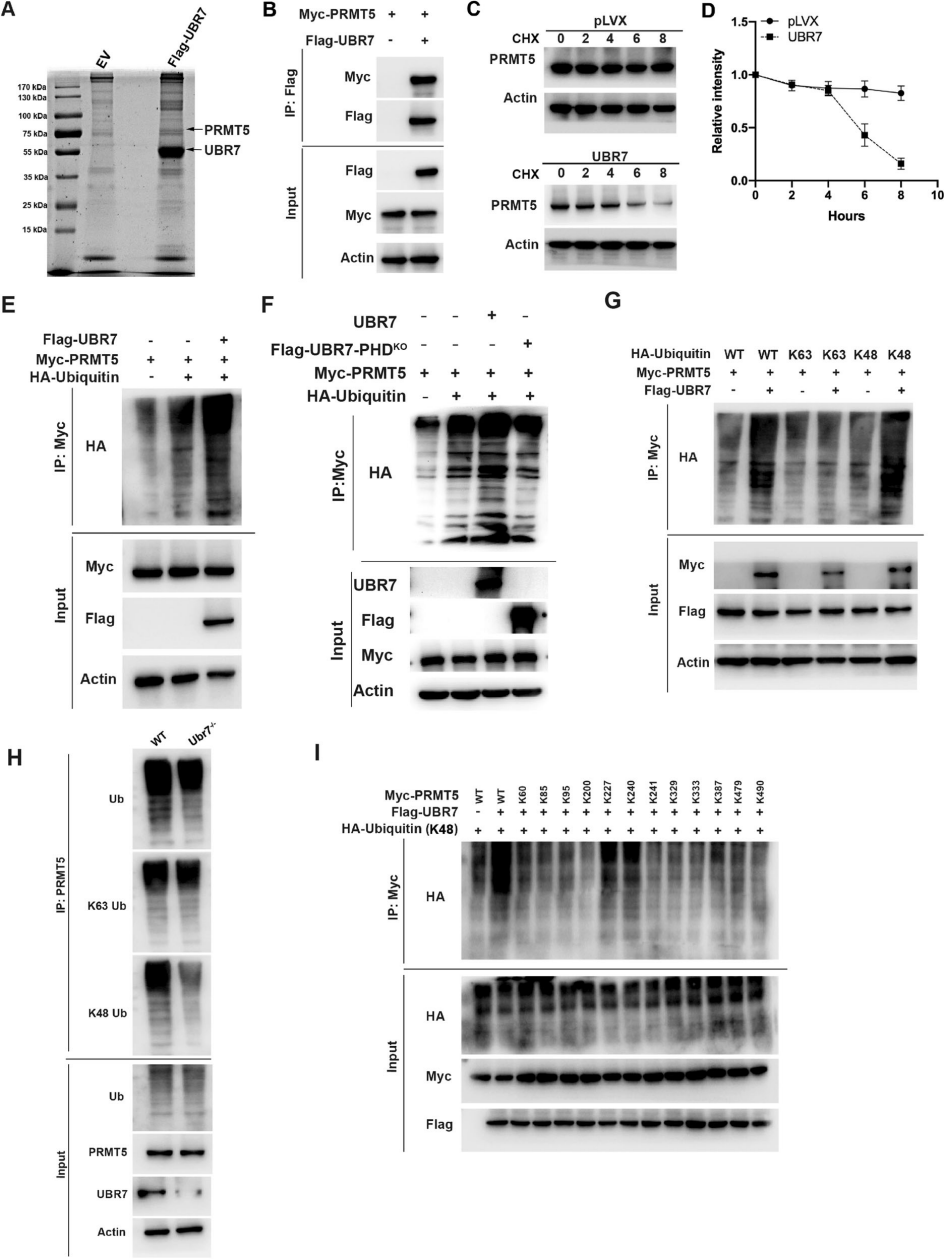
UBR7 degrades PRMT5 through K48 ubiquitination. **A** HEK293T cells overexpressed UBR7, and the protein interacting with UBR7 was identified by mass spectrometry. **B** The interaction between UBR7 and PRMT5 was verified by immunoprecipitation in HEK293T cells. **C** CHX (Cycloheximide) treatment of HEK293T cells overexpressing UBR7, and Western blot detection of PRMT5 protein level changes. **D** Quantification of the PRMT5 protein level in (**C**). **E** HEK293T cells overexpressing UBR7 were immunoprecipitated to detect PRMT5 ubiquitination levels. **F** HEK293T cells with overexpressing UBR7 or UBR7-PHD^KO^ were immunoprecipitated to detect PRMT5 ubiquitination levels. **G** HEK293T cells overexpressed UBR7, PRMT5 and K63 or K48, and immunoprecipitation was used to detect PRMT5 ubiquitination levels. **H** Ductal epithelial cells of the pancreas were extracted from 8-week-old WT and *Ubr7*^–/–^ mice to detect the ubiquitination level of PRMT5. **I** A lysine point mutant of PRMT5 was constructed, and the ubiquitination level of PRMT5 was detected by immunoprecipitation.

To further explore the mechanism of UBR7 in PDAC, we overexpressed UBR7 in HEK293T cells and performed mass spectrometry to identify the proteins that bind to it (Fig. 6A). Mass spectrometry results showed that PRMT5 had the largest number of peptides among the proteins that bind to UBR7 (Supplementary Fig. S5A). The immunoprecipitation results also showed that UBR7 and PRMT5 bound to each other (Fig. 6B and Supplementary Fig. S5B). Furthermore, we found that overexpression of UBR7 significantly reduced PRMT5 protein levels (Supplementary Fig. S5C). Knockdown of UBR7 significantly increased PRMT5 protein levels (Supplementary Fig. S5D), and PRMT5 protein levels in KC;*Ubr7*^–/–^ pancreatic cells also significantly increased (Supplementary Fig. S5E). We also determined the mRNA expression level of PRMT5 in UBR7-overexpressing, UBR7-knockdown cells and mouse pancreatic tissues, and the results showed that overexpression or knockdown of UBR7 did not affect the mRNA level of PRMT5 (Supplementary Fig. S5F–H).

The results of the protein stability experiments showed that overexpression of UBR7 significantly reduced the protein stability of PRMT5 (Fig. 6C, D). In addition, we also found that UBR7 significantly increased the ubiquitination level of PRMT5 (Fig. 6E). It has been reported that the plant homeodomain (PHD) finger of UBR7 is critical for its association with E2 UbcH6 and subsequent transfer of ubiquitin to its substrate histone H2B [25]. We used the recombinant human UbcH (E2) Enzyme Set Protein kit to identify the E2 enzyme that interacts with UBR7 as UbcH6. In view of the importance of the PHD structure for UBR7 [25], we knocked out the PHD sequence of UBR7 and then tested whether it had an effect on the ubiquitination of PRMT5. The results showed that the PHD structure of UBR7 was critical for ubiquitination of PRMT5 (Fig. 6F). In addition, we examined whether UBR7 transfers ubiquitin to its substrate histone H2BK120 and plays a role in pancreatic cancer cells. It was found that the expression of UBR7 was significantly positively correlated with H2BK120Ub (Supplementary Fig. S5D, S5I). However, after we simultaneously overexpressed UBR7 and H2BK120R in panc-1 GEM cells, we found no difference in viability compared with cells that only overexpressed UBR7 (Supplementary Fig. S5J). This indicates that UBR7 plays a role in ubiquitination of H2B in pancreatic cancer cells, but may not rely on ubiquitination of H2B to function. Furthermore, in vivo and in vitro ubiquitination experiments revealed that UBR7 ubiquitinated PRMT5 through the K48 ubiquitin linkage (Fig. 6G, Supplementary Fig. S6A, S6B). Compared with WT, the ubiquitination level of the PRMT5 K48 ubiquitin connection mode was significantly reduced in the *Ubr7*^–/–^ mouse pancreas cells (Fig. 6H). Finally, we also predicted the PRMT5 ubiquitination site and constructed point mutants (Supplementary Fig. S6C). Coimmunoprecipitation results showed that the ubiquitinated PRMT5 sites of UBR7 were K227 and K240 (Fig. 6I). The K227R and K240R mutants of PRMT5 were constructed, and the results showed that the levels of K227R and K240R mutants of UBR7 ubiquitinated PRMT5 were significantly reduced (Supplementary Fig. S6D). The above results indicate that UBR7 degrades PRMT5 through ubiquitination and that the sites where K48 ubiquitin connects to PRMT5 are K227 and K240.

### UBR7 mediates gemcitabine resistance in pancreatic cancer by regulating PRMT5 expression

**Fig. 7:**
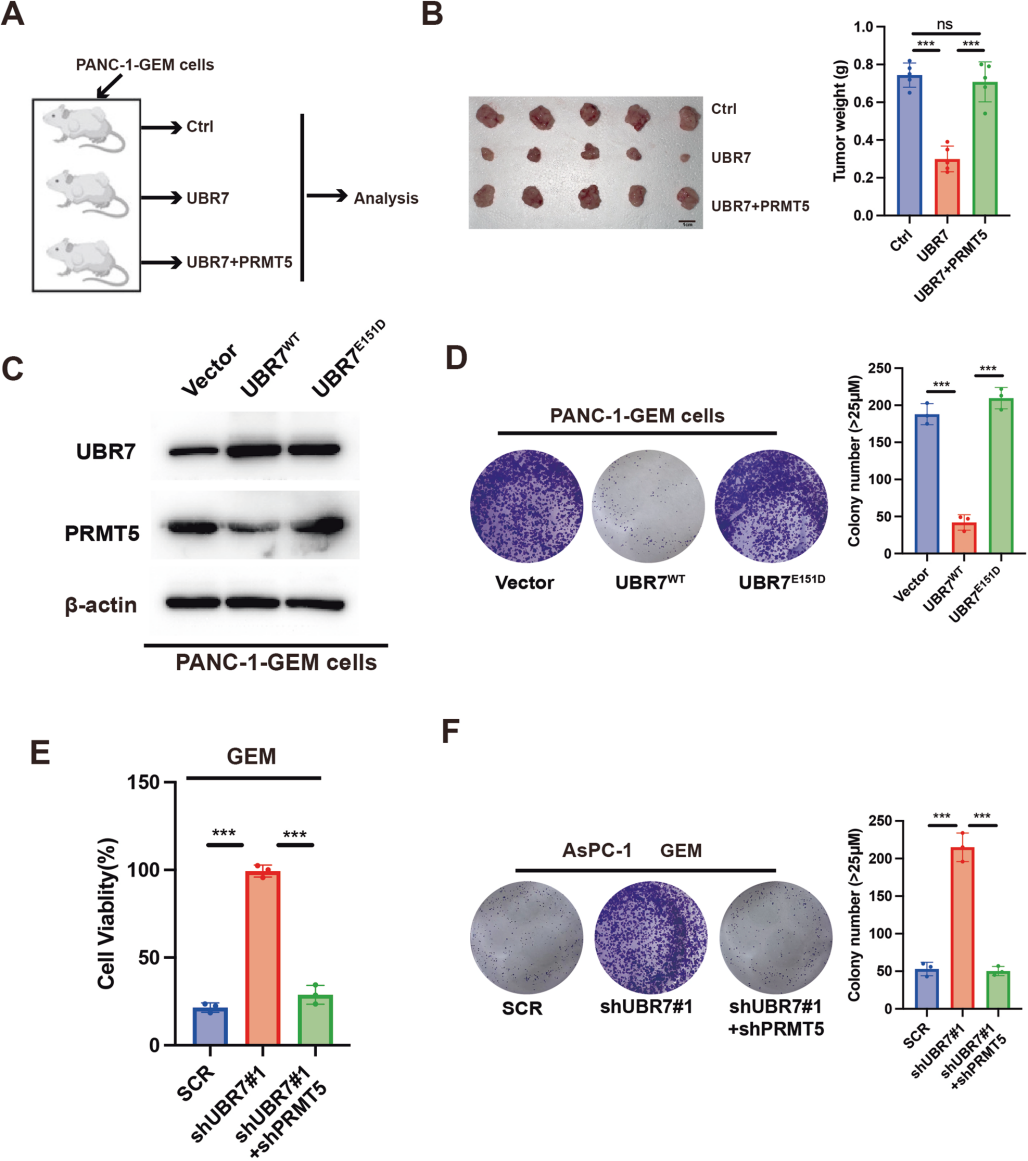
UBR7 mediates gemcitabine resistance in pancreatic cancer by regulating PRMT5 expression. **A** PANC-1-GEM cells with vector, overexpressing UBR7 or simultaneously overexpressing UBR7 and PRMT5 transplanted subcutaneously into nude mice. **B** The transplanted tumors of the three groups of mice were dissected, and the tumor weights were counted one month after inoculation. **C** WB detected the expression of UBR7 and PRMT5 in PANC-1-GEM cells transfected with UBR7^WT^ and UBR7^E151D^ mutant. **D** Clone formation assay to detect the proliferation ability of cells transfected with UBR7^WT^ and UBR7^E151D^ mutant. MTT and colony formation were used to detect the cell viability (**E**) and proliferation (**F**) of UBR7 knockdown or simultaneous knockdown of UBR7 and PRMT5 in Aspc-1 cells. *p < 0.05, **p < 0.01, ***p < 0.001, ns=no difference. Error bars represent the standard error for three technical replicates.

In order to prove whether UBR7 deletion promoting the progression and drug resistance of pancreatic cancer was related to the regulation the expression of PRMT5, we overexpressed UBR7 and simultaneously overexpressed UBR7 and PRMT5 in PANC-1-GEM cells, and then transplanted the cells subcutaneously into nude mice (Fig. 7A). We found that overexpression of UBR7 could effectively reduce the size of tumors, while the tumor after simultaneous overexpression of PRMT5 weight was larger (Fig. 7B). Then, we constructed the catalytic mutant of UBR7, UBR7^E151D^. Western Blot demonstrated that increased PRMT5 expression after UBR7 loss of function. (Fig. 7C). Clone formation experiments further confirmed that PRMT5 could reverse the GEM sensitivity of PANC-1-GEM cells caused by UBR7 overexpression (Fig. 7D). Next, we knocked down the expression of PRMT5 in UBR7-knockdown AsPC-1 cells and detected the cell viability after 48 h of GEM treatment. The results showed that knocking down PRMT5 could reverse the GEM resistance of PANC-1-GEM cells caused by UBR7 deletion (Fig. 7E). Clone formation experiments further confirmed this result in AsPC-1 cells (Fig. 7F). Taken together, these results indicate that UBR7 regulates the progression and GEM resistance of pancreatic cancer by regulating the expression of PRMT5.

### Blocking the degradation of PRMT5 by UBR7 deficiency increases glycolysis in PDAC cells

**Fig. 8:**
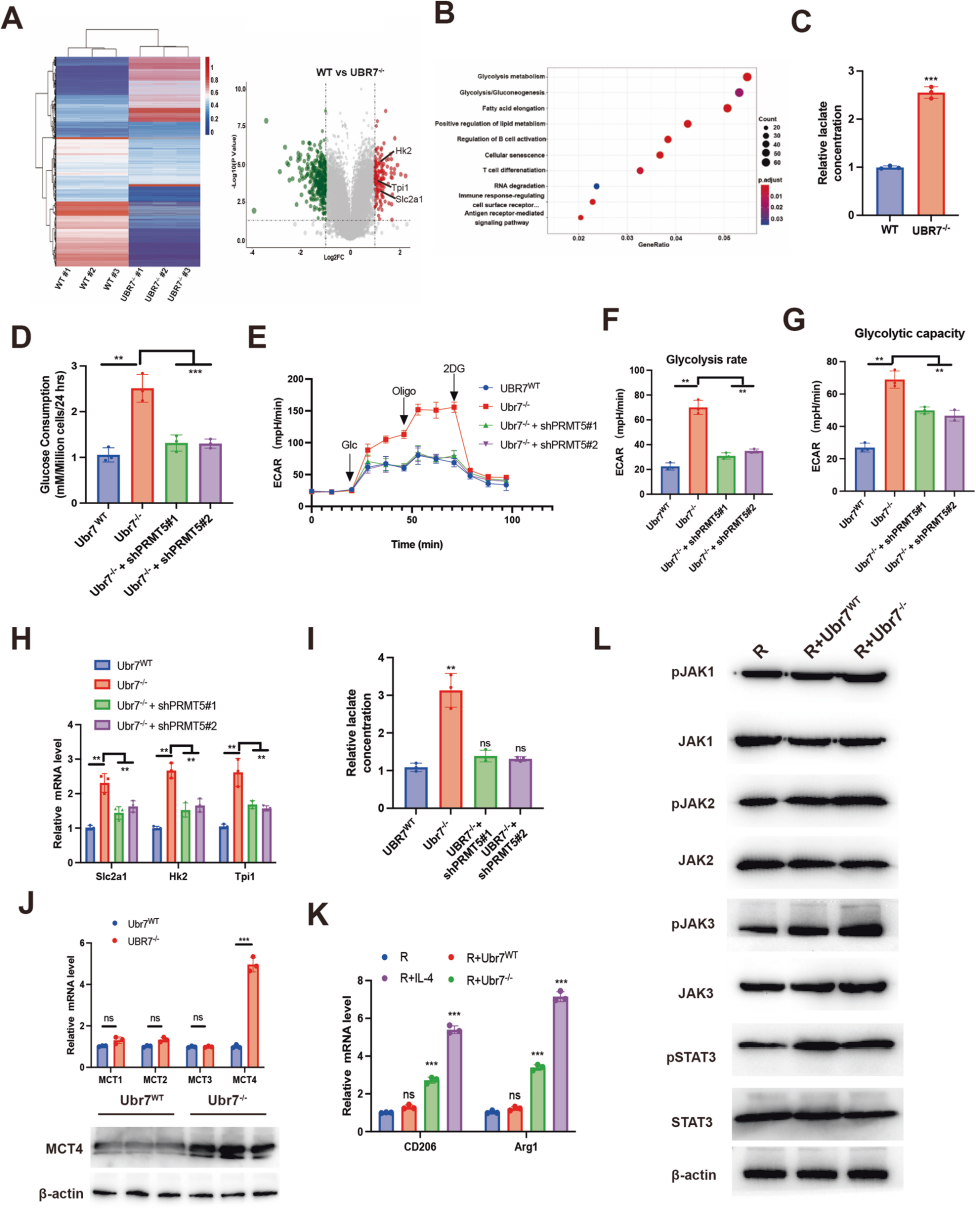
Blocking the degradation of PRMT5 by UBR7 deficiency increases glycolysis in PDAC cells. **A** RNA sequencing of ductal epithelial cells of the pancreas from WT and *Ubr7*^–/–^ mice. A heatmap and volcano map were plotted to analyze the differentially expressed genes. **B** Functional cluster analysis of the differentially expressed genes in (**A**). **C** The lactate levels of pancreatic tissues in WT and *Ubr7*^–/–^ mice. **D** Cells were transduced with Ad or Ad-shPRMT5. Glucose consumption of WT and Ubr7-deficient pancreatic ductal epithelial cells. **E** ECAR of WT and UBR7-deficient pancreatic ductal epithelial cells transduced with Ad or Ad-shPRMT5. Glycolysis rate (**F**) and Glycolytic ability (**G**) were measured in transduced with Ad or Ad-shPRMT5 Glucose consumption of WT and Ubr7-deficient pancreas ductal epithelial cells. **H** Real-time PCR analysis of Slc2a1, Hk2 and Tpi1 in transduced with Ad or Ad-shPRMT5 in WT and Ubr7-deficient pancreas ductal epithelial cells. **I** The lactate levels in transduced with Ad or Ad-shPRMT5 in WT and Ubr7-deficient pancreas ductal epithelial cells. **J** RNA expression levels of MCT1-4 and protein expression levels of MCT4 in pancreatic tissues of WT and *Ubr7*^–/–^ mice. **K** M2 marker detection after mouse macrophages RAW264.7 (R) were cultured alone, WT or *UBR7*^–/–^ mouse pancreatic cells were co-cultured with R, and R received IL4 induction. **L** Mouse macrophages RAW264.7(R) were cultured alone, WT or *UBR7*^–/–^ mouse pancreatic cells were co-cultured with R and R was induced by IL4, and the JAK-STAT3 signaling pathway was detected. *p < 0.05, **p < 0.01, ***p < 0.001, ns = no difference. Error bars represent the standard error for three technical replicates.

We performed RNA sequencing and compared the gene expression profiles from wild-type (WT) mice and *Ubr7*^−/−^ mouse pancreas tissues and observed the upregulation of glycolytic genes in UBR7-deficient cells (Fig. 8A, B). We detected the lactate levels in the pancreatic tissues of two groups of mice and found that the lactate levels in *Ubr7*^−/−^ mice were significantly increased, providing conditions for glycolysis (Fig. 8C). To investigate whether the elevated glycolysis rate in UBR7-deficient cells is dependent on PRMT5, we knocked out PRMT5 in UBR7-deficient cells. It is worth noting that knocking down PRMT5 offset the upregulation of the glucose consumption rate (Fig. 8D), glycolysis rate (Fig. 8E–G) and glycolytic gene transcription (Fig. 8H) in UBR7-deficient cells. Similarly, we examined lactate levels and showed that knocking down PRMT5 counteracted the high lactate levels in UBR7-deficient cells (Fig. 8I). Lactic acid has two main ways to transmit signals to cells: mediated by transporters(MCT) or sodium-dependent co-transports; and receptors(GPR). So, we examined the expression of MCTs in pancreatic tissues of WT and *Ubr7*^−/−^ mice, and found that the expression of MCT4 was significantly elevated in *Ubr7*^−/−^ mice (Fig. 8J). It has been reported that MCT4 may play a key role in maintaining the tumor immune microenvironment (TIME) through metabolic reprogramming, including inducing macrophage polarization [26, 27]. We co-cultured WT and *UBR7*^−/−^ mice pancreatic cells with mouse macrophage RAW264.7(R), respectively, and found that *Ubr7*^−/−^ mice pancreatic cells could promote M2 macrophage polarisation through the JAK/STAT3 signaling pathway (Fig. 8K, L). Therefore, all these results indicated that blocking the degradation of PRMT5 by UBR7 deficiency increases glycolysis in PDAC cells to maintain the optimal inhibitory function.

### UBR7 deletion impacts the immune response in PDAC

**Fig. 9:**
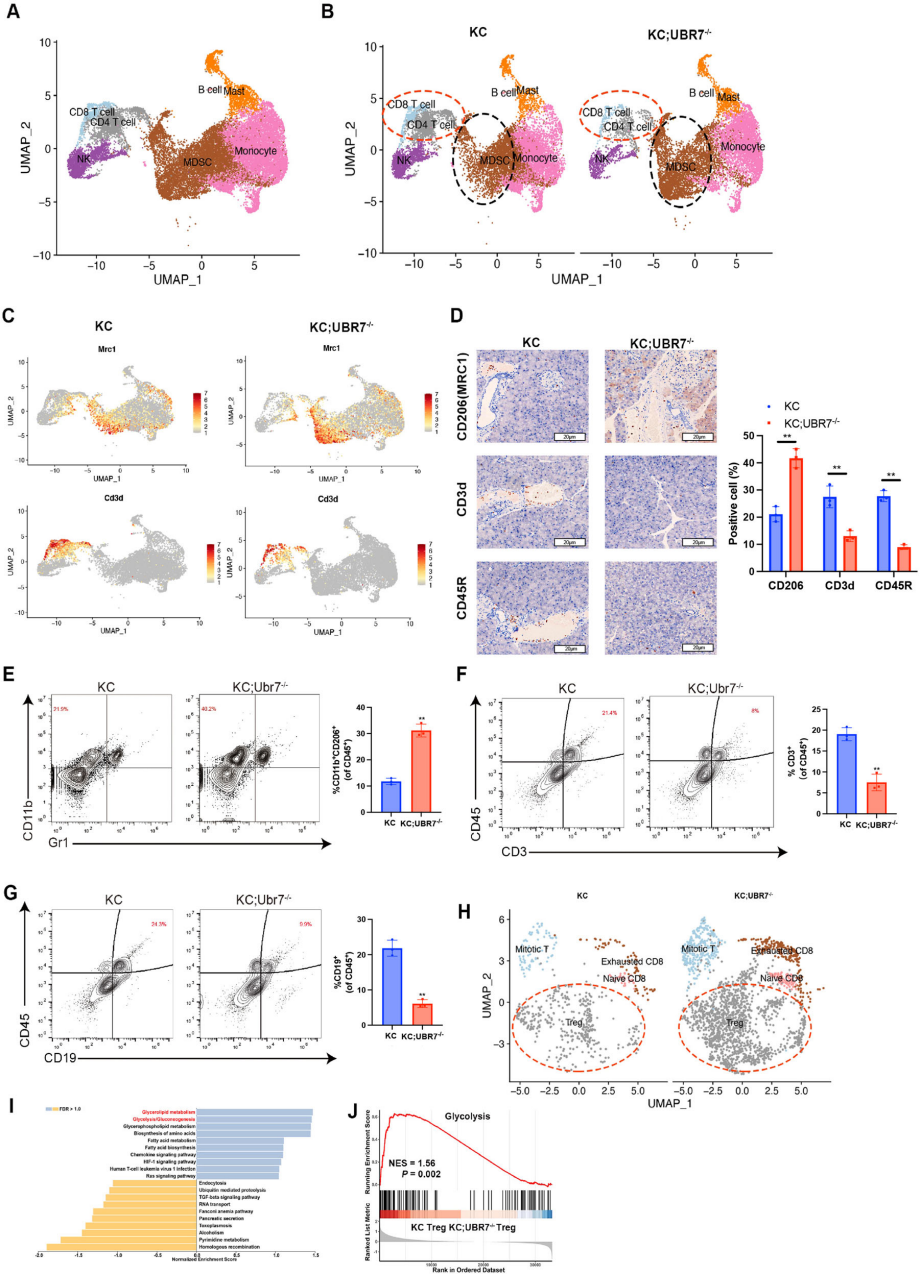
UBR7 deletion impacts the immune response in PDAC. **A** Unfractionated live cell mixtures from KC and KC;*Ubr7*^*–/–*^ mouse pancreatic tumors were subjected to scRNA-seq analysis, and the functional clusters of the cells were analyzed. **B** Comparison of the distribution of cell clusters in pancreatic cancer tissues of KC and KC;*Ubr7*^*–/–*^ mice. **C** The standardized expression levels of myeloid-2/MDSC, T-cell and B-cell marker genes in pancreatic cancer tissue of KC and KC;*Ubr7*^*–/–*^ mice; (**D**) representative immunohistochemical staining images and quantification of CD206 (MRC1) in MDSCs, CD3 in T cells and CD45R in B cells in pancreatic cancer tissue of KC and KC;*Ubr7*^*–/–*^ mice. **E–G** Flow cytometry was performed to detect the percentage of CD11b^+^ CD206^+^ bone marrow cells, CD3^+^ T cells and CD19^+^ B cells in CD45^+^ immune cells in pancreatic cancer tissues of KC and KC;*Ubr7*^*–/–*^ mice. **H** Perform cell cluster clustering on lymphocytes in KC and KC;*Ubr7*^*–/–*^ mouse pancreatic cancer tissues. **I** Functional clustering of differentially expressed genes in Treg cells in KC and KC;*Ubr7*^*–/–*^ mouse pancreatic cancer tissues. **J** Gene set enrichment analyses (GSEA) of DEGs between KC and KC;*Ubr7*^*–/–*^ mouse Treg cells in pancreatic cancer tissue revealed a significant enrichment of glycolysis-associated genes. **p < 0.01. Error bars represent the standard error for three technical replicates. The scale bar is 20 μm in (**D**).

Pancreatic cancer is in an immunosuppressive microenvironment [28]. Therefore, we further explored the potential effect of UBR7 depletion on the PDAC tumor microenvironment. We used scRNA-seq to analyze the unfractionated CD45^+^ cell mixtures of KC and KC;*Ubr7*^*–/–*^ tumor tissues (Fig. 9A). The myeloid cells, T cells, and B cells in KC;*Ubr7*^*-/-*^ tumors were significantly changed (Fig. 9B, Supplementary Fig. S7A). KC;*Ubr7*^*-/-*^ tumors showed an increase in the number of myeloid-derived suppressor cells (MDSCs) and a decrease in T/B lymphocytes (Fig. 9B, C), which was related to the accelerated progression of KC;*Ubr7*^−/−^ tumors. Immunohistochemical staining of CD206, CD3 and CD45R in two advanced PDAC slices from KC and KC;*Ubr7*^*–/–*^ mice further confirmed the increased M2 macrophages and decreased T lymph in KC;*Ubr7*^*–/–*^ tumors (Fig. 9D). Consistent results were also observed in the flow cytometry analysis of immune cell composition (increased M2 macrophages and decreased T/B cells) in KC;*Ubr7*^*-/-*^ tumors compared with KC tumors (Fig. 9E–G).

To further analyze the effect of exhausted UBR7 on immune cells in the microenvironment of pancreatic cancer tissues, we performed a refined subpopulation analysis of T cells. Among them, we found that compared with KC tumor tissue, Treg cells in KC;*Ubr7*^*-/-*^ tumor tissue were significantly increased (Fig. 9H, Supplementary Fig. S7B). This finding suggested that it was possible that Tregs increased the immunosuppressive microenvironment in KC;*Ubr7*^*–/–*^ tumor tissues. Further functional cluster analysis of the differentially expressed genes in Treg cells in KC and KC; *Ubr7*^*–/–*^ tumor tissues revealed that the glycolysis-related genes of Treg cells in KC;*Ubr7*^*–/–*^ tumor tissues were significantly increased (Fig. 9I). The additional gene set enrichment analysis (GSEA) also confirmed the specific enrichment of the glycolytic pathway of Treg cells in the tumor (Fig. 9J). In addition, we also found that CD4^+^ and CD8^+^ T cells secreted PD-1, granzyme B, CD107a and TNF-α levels in tumor tissues of KC;*Ubr7*^*–/–*^ mice were significantly reduced (Supplementary Fig. S8A, S8B). These results indicate that depleted UBR7 may promote PDAC tumorigenesis by changing the antitumor immune microenvironment.

### Inhibition of PRMT5 effectively reverses GEM resistance in PDAC cells

We found that depleted UBR7 stabilized PRMT5 protein, increased glycolysis in cells and promoted T-cell depletion. We further explored whether the combination of a PRMT5 inhibitor (DS-437) and GEM therapy alleviated the growth of GEM-resistant tumors with low UBR7 expression. Pano2-shUBR7 cells were subcutaneously inoculated into C57BL/6 mice to obtain tumor tissues and then treated with DS-437 or GEM (Supplementary Fig. S9A). Deletion of UBR7 increased PDAC cell insensitivity to GEM (Supplementary Fig. S9B, S9C). In addition, DS-437 combined with GEM treatment further reduced the tumor size compared with that in the GEM group (Supplementary Fig. 9B, C). We further observed that inhibition of PRMT5 restored the function of exhausted CD8^+^ T cells, characterized by a decrease in the percentage of PD-1^+^ CD8^+^ T cells (Supplementary Fig. S9D, S9E). In summary, these results indicate that inhibition of PRMT5 activity restores the function of exhausted CD8^+^ T cells and effectively inhibits pancreatic carcinogenesis and GEM resistance caused by UBR7 depletion in PDAC cells.

## Discussion

Studies have found that UBR7 monoubiquitinated histone H2B inhibits triple-negative breast cancer tumorigenesis and metastasis [29]. UBR7 stabilizes the phosphoribosyl pyrophosphate synthetase (PRPS) catalytic subunits by mediating the degradation of PRPS-related proteins (negative regulators of PRPS) caused by polyubiquitination and then regulates nucleotide metabolism in acute lymphoblastic leukemia [30]. The mechanism of UBR7 in tumor resistance, especially in gemcitabine-resistant pancreatic cancer, has rarely been reported. In this study, analysis of chromatin accessibility and transcriptional regulation in gemcitabine-resistant or gemcitabine-sensitive PDAC-PDX tissues identified that UBR7 was significantly reduced in PDAC-PDX tissues that were resistant to gemcitabine. Furthermore, we found that depletion of UBR7 promoted pancreatic cancer progression and an immunosuppressive microenvironment. Mechanistically, UBR7 also monoubiquitinated histone H2B in pancreatic cancer cell lines, but did not function. Depletion of UBR7 increased PRMT5 protein stability, which in turn increased glycolysis in pancreatic cancer cells and Tregs. Finally, the use of PRMT5 inhibitors markedly increased the gemcitabine sensitivity of UBR7-depleted PDACs.

PRMT5 modulates the expression of broad-spectrum target genes by modifying chromatin structure or transcription mechanisms. In addition, certain tumor suppressors, such as the metastasis suppressor Nm23, can be epigenetically silenced by PRMT5 [31]. Therefore, PRMT5 mainly functions as a tumor-promoting factor. PRMT5 has also recently been recognized as an important regulator of cell metabolism [18, 21, 32]. PRMT5 regulates glycolysis in pancreatic cancer through the FBW7/cMyc axis and promotes the occurrence of pancreatic cancer tumors [18]. Recently, similar metabolic effects have been observed in murine and/or human T lymphocytes [21]. Knockout of PRMT5 in human transformed human Jurkat T cells resulted in reduced glycolytic metabolism and reduced oxidative phosphorylation [21]. In this study, we found that depleted UBR7 promoted glycolysis in PDAC cells. Further mechanistic studies have found that UBR7 ubiquitinates and degrades PRMT5. In PDAC cells depleted of UBR7, the stability of PRMT5 protein was increased, which in turn increased the expression of glycolysis-related genes and caused an increase in glycolysis in PDAC cells.

The tumor microenvironment in pancreatic cancer is highly immunosuppressive and is often referred to as an immunological “cold” tumor. Therefore, targeting and restoring the patient’s immune system through engineered biomaterials provides an attractive treatment method [33, 34]. The regulation of tumor-related immunosuppressive cells, such as tumor-associated macrophages (TAMs), MDSCs and regulatory T cells (Tregs), can effectively activate the innate and adaptive immune response against pancreatic cancer [35]. Studies have shown that glycolysis regulates Treg induction and immunosuppressive functions by controlling the Foxp3 splice variant containing exon 2 (Foxp3-E2) through the glycolytic enzyme enolase-1 [3]. The migration of Tregs to inflamed tissues is also dependent on glucokinase (GCK) in glycolysis [36]. In tumor tissue, Tregs induce cellular senescence and suppress effector T cells by depleting glucose, thereby increasing the immunosuppressive tumor microenvironment [37]. In this study, we performed functional enrichment analysis of differentially expressed genes in Tregs in KC and KC;*UBR7*^−/−^ pancreatic cancer tissues and showed that depletion of UBR7 increased glycolysis in Tregs, which was not experimentally verified. In our follow-up research, we will continue to explore the relevant mechanism by which UBR7 regulates Tregs glycolysis and look forward to revealing this regulatory mechanism.

In conclusion, we found that UBR7 is depleted in gemcitabine-resistant PDAC and further found that depleted UBR7 stabilizes PRMT5 to increase pancreatic cancer cell and Treg cell glycolysis, leading to pancreatic cancer progression and an immunosuppressive microenvironment. The mechanism of UBR7-PRMT5 regulation of glycolysis elucidated in this study may provide a potential explanation for the drug resistance and immunosuppressive microenvironment of pancreatic cancer. Furthermore, this study provides a rationale for a clinical regimen that blocks PRMT5-mediated glycolysis in patients with gemcitabine-resistant UBR7-depleted pancreatic cancer.

## Supplementary information

Supplementary figure

Original Data

## Data Availability

The data that support the findings of this study are available from the corresponding author upon reasonable request.
