# Supplementary material for: The interaction between UBR7 and PRMT5 drives PDAC resistance to gemcitabine by regulating glycolysis and immune microenvironment
Source: Cell Death Dis. 2024 Oct 18;15(10):758. doi: 10.1038/s41419-024-07145-z (PMC11489413; doi:10.1038/s41419-024-07145-z)
Supplement: Supplementary file 1 — Supplementary figure [file 41419_2024_7145_MOESM1_ESM.docx]

**Supplementary figure and figure legends**


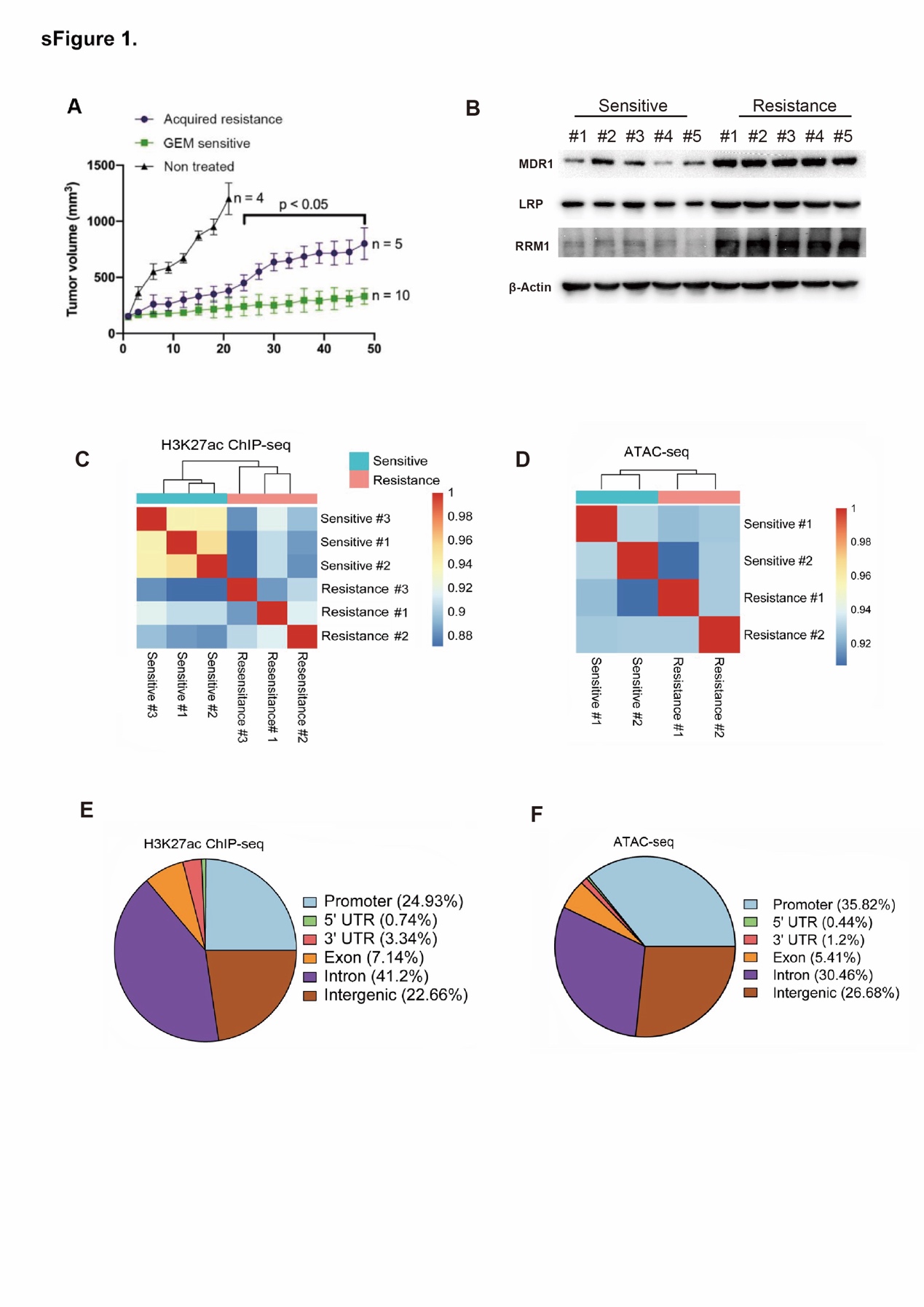


**Supplementary Figure 1.** **A,** Tumor growth in PDX mouse in Figure 1A. **B,** The traditional resistance markers MDR1, LRP and RRM1 in sensitive and resistant tumor tissues. **C** and **D**, Read count correlation of ChIP-seq (C) and ATAC-seq (D) peaks in sensitive and resistant tumor tissues. **E** and **F,** Pie chart showing the genomic annotations of the union of 98,145 and 26,402 ChIP-seq (E) and ATAC-seq (F) peaks, respectively.


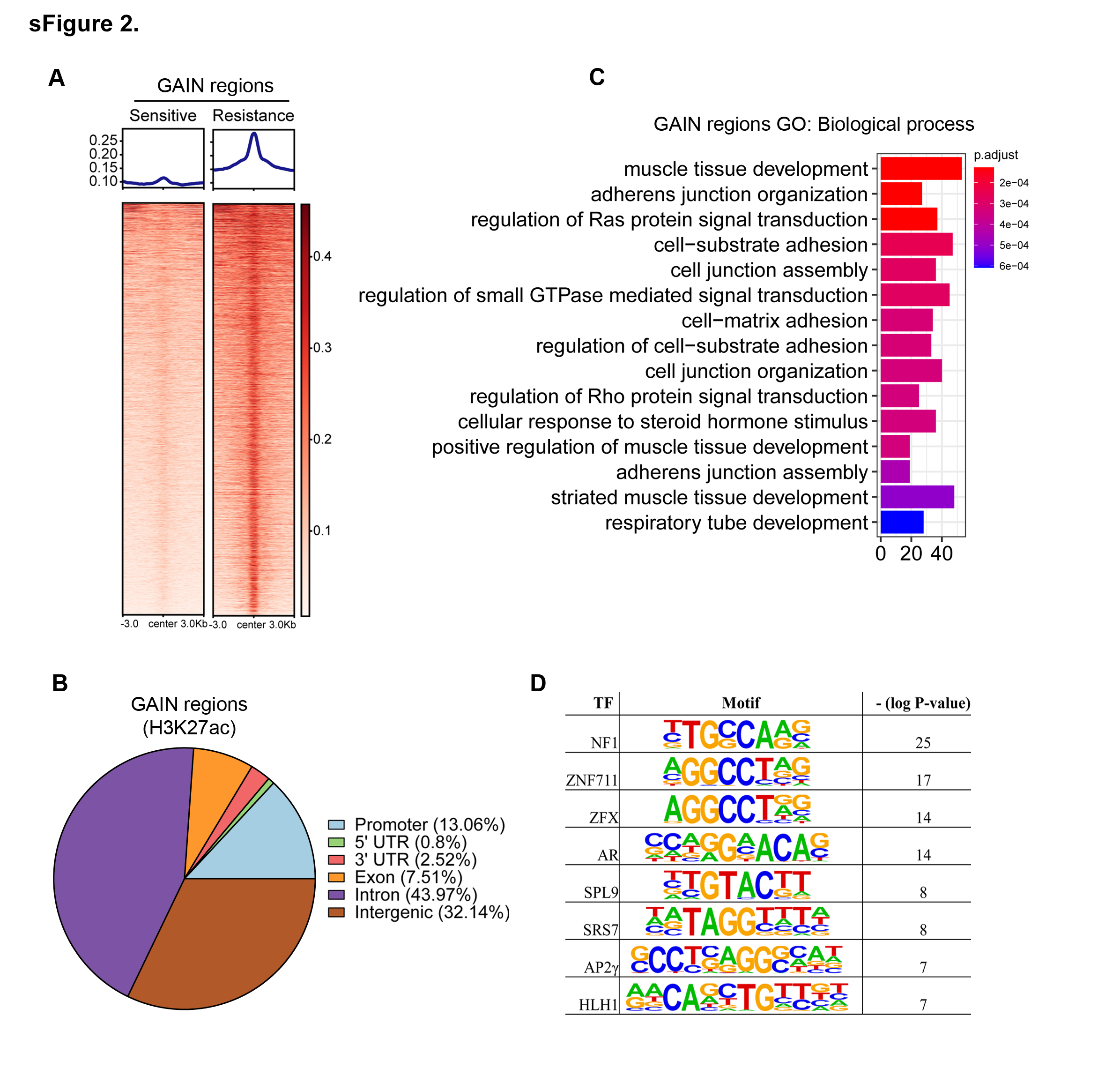


**Supplementary Figure 2.** **A,** Heatmap representation of GAIN regions based on H3K27ac occupancy in PDX models. **B,** Pie chart showing the genomic annotations of GAIN regions according to the location of a given peak. **C,** GO analysis indicated the GAIN peaks-related signaling pathway in resistant samples. **D,** Motif enriched in GAIN regions.


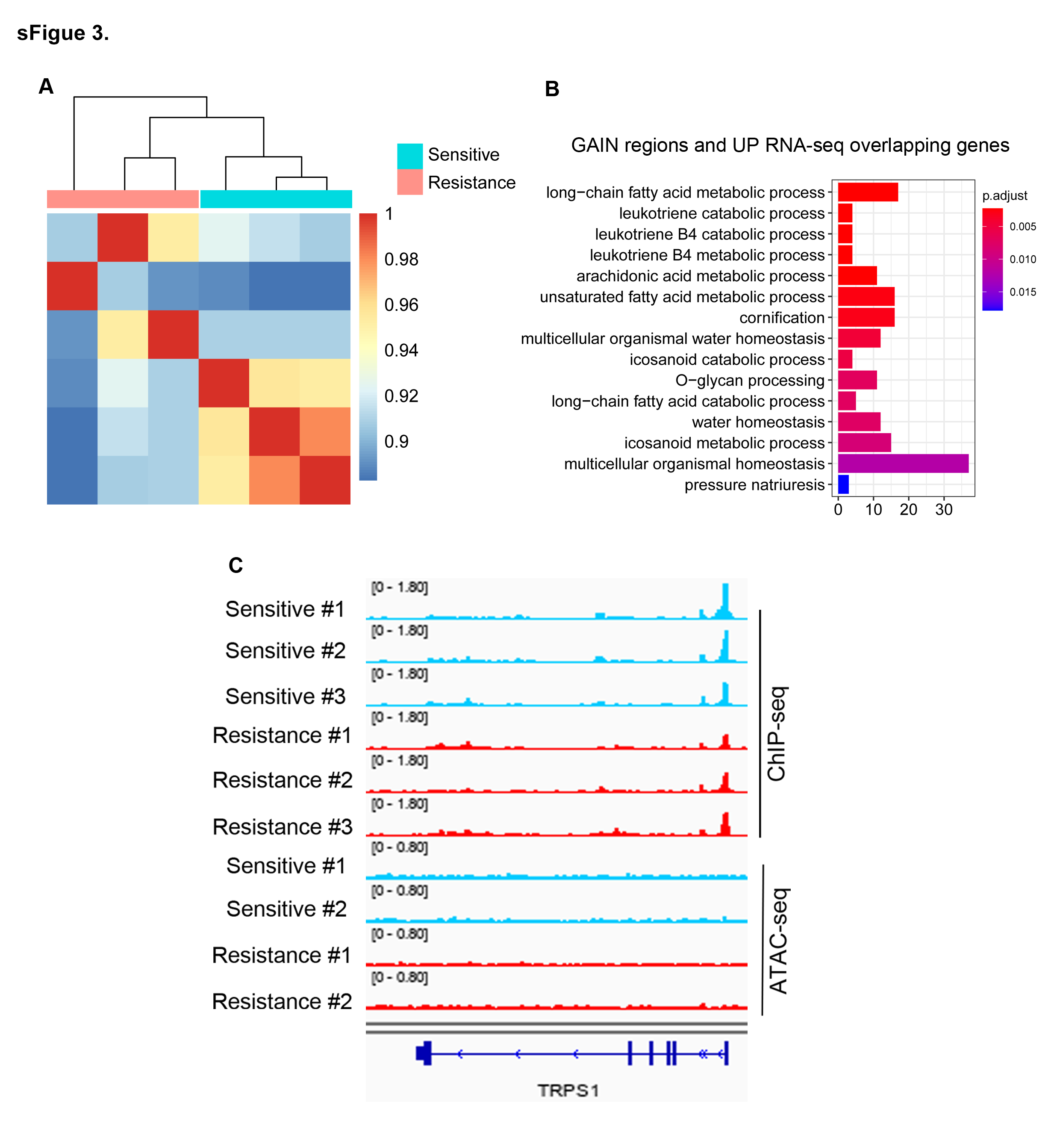
**Supplementary Figure 3. A,** Heat map show the correlation of the sensitive and resistant tumor samples. **B,** GO analysis was performed on the GAIN peaks-associated genes that were also upregulated in the RNA-seq analysis. **C,** H3K27ac and ATAC signals at the TRPS1 gene locus.


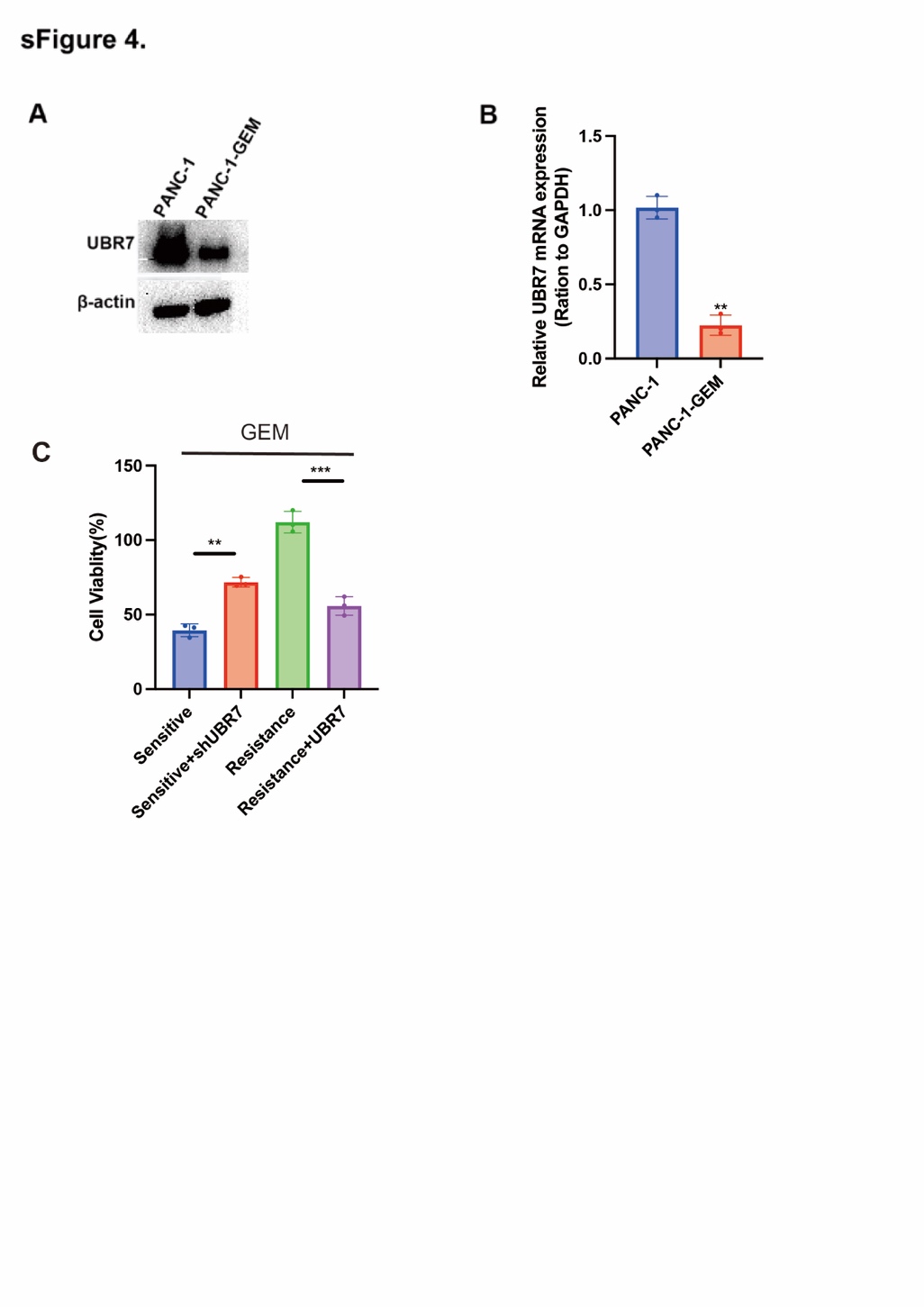


**Supplementary Figure 4. A** and **B,** UBR7 protein expression level (A) and mRNA level (B) in human pancreatic drug-resistant cell line PANC-1-GEM and sensitive cell PANC-1. **C,** Down-regulates the expression of UBR7 in cells in the sensitive group and up-regulates the expression of UBR7 in cells in the resistant group. Gemcitabine (1uM) was applied to the above cells, and then the cell viability was detected.


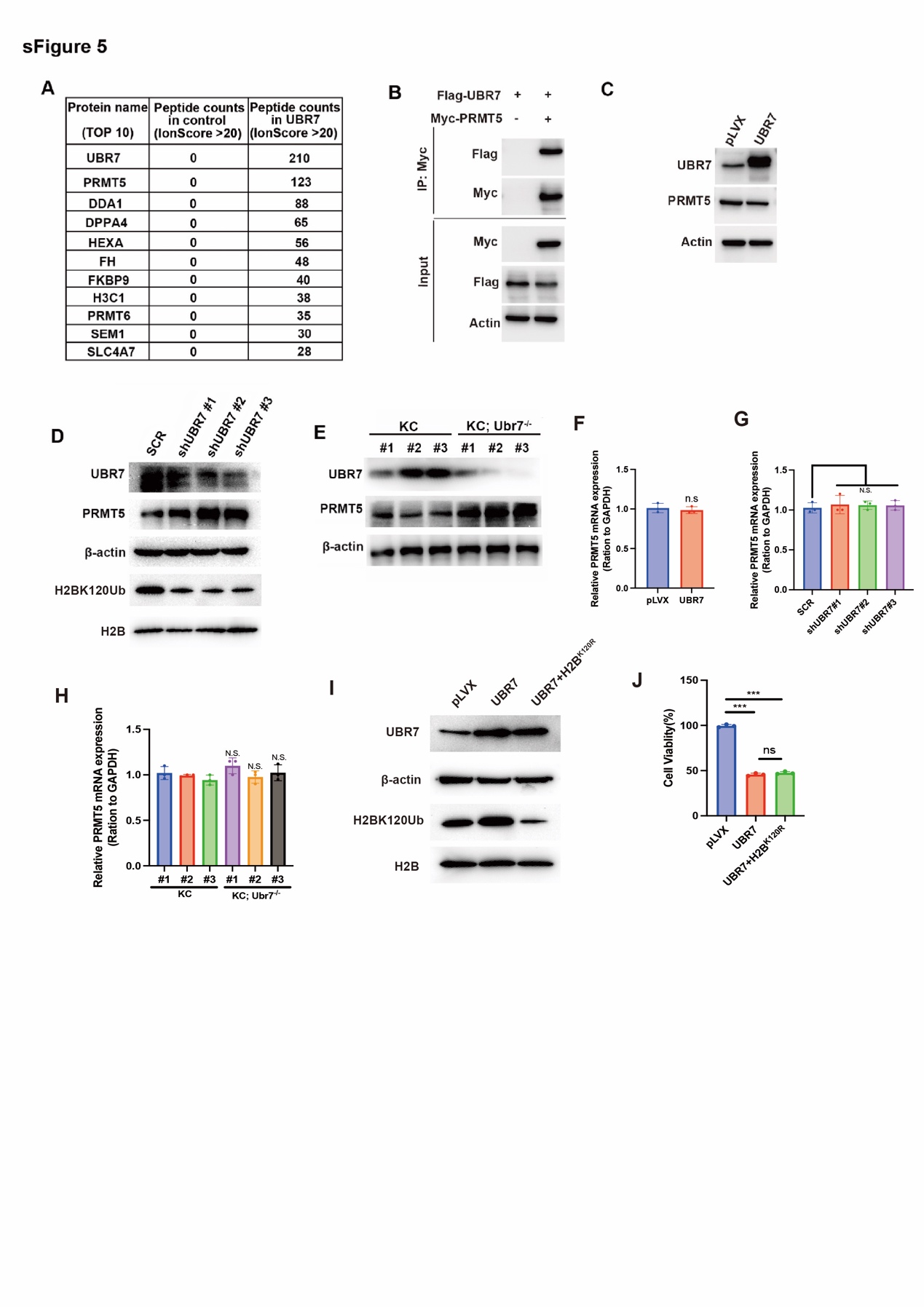


**Supplementary Figure 5. A**, Mass spectrometry identified UBR7 binding proteins (Top10). **B,** The interaction between UBR7 and PRMT5 was verified by immunoprecipitation in HEK293T cells; **C,** HEK293T cells overexpressing UBR7, and PRMT5 protein was detected by Western blot. **D,** In BxPC-3 cells knocked down UBR7, the expression level of PRMT5 and H2BK120Ub protein was detected by Western blot. **E,** The PRMT5 protein expression level in pancreas ductal epithelial cells from KC and KC;*Ubr7^-/-^* mice were measured by Western blot. **F,** In Panc-1 cells overexpressed UBR7, the expression level of PRMT5 mRNA was detected by qRT-PCR. **G,** In AsPC-1 cells knocked down UBR7, the expression level of PRMT5 mRNA was detected by qRT-PCR. **H**, qRT-PCR detects the expression level of PRMT5 mRNA in pancreas ductal epithelial cells from KC and KC;*Ubr7^-/-^* mice. **I**, Overexpression of UBR7 or both UBR7 and H2BK120R in panc-1 GEM cells, the expression of UBR7 and H2BK120Ub was detected. **J**, Cell viability of panc-1 GEM cells overexpressing UBR7 or both UBR7 and H2BK120R was tested after 48 h of GEM induction. N.S. = No difference. Data are presented as mean ± standard error.


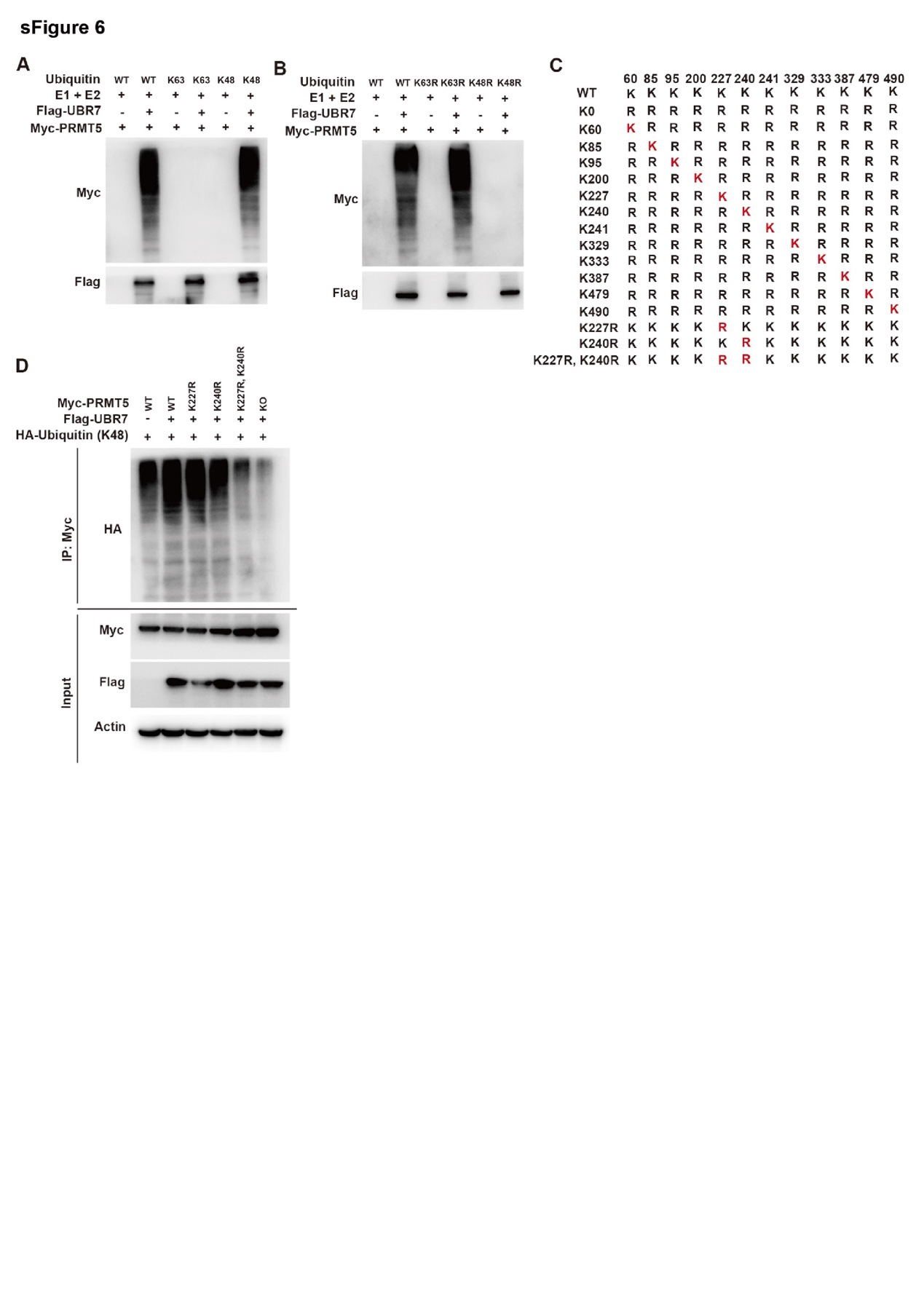


**Supplementary Figure 6. A** and **B,** In vitro ubiquitination detection of the ubiquitination level of PRMT5 by UBR7. **C**, Design and construction of a point mutant of PRMT5. **D,** UBR7 ubiquitinates the lysine site of PRMT5.


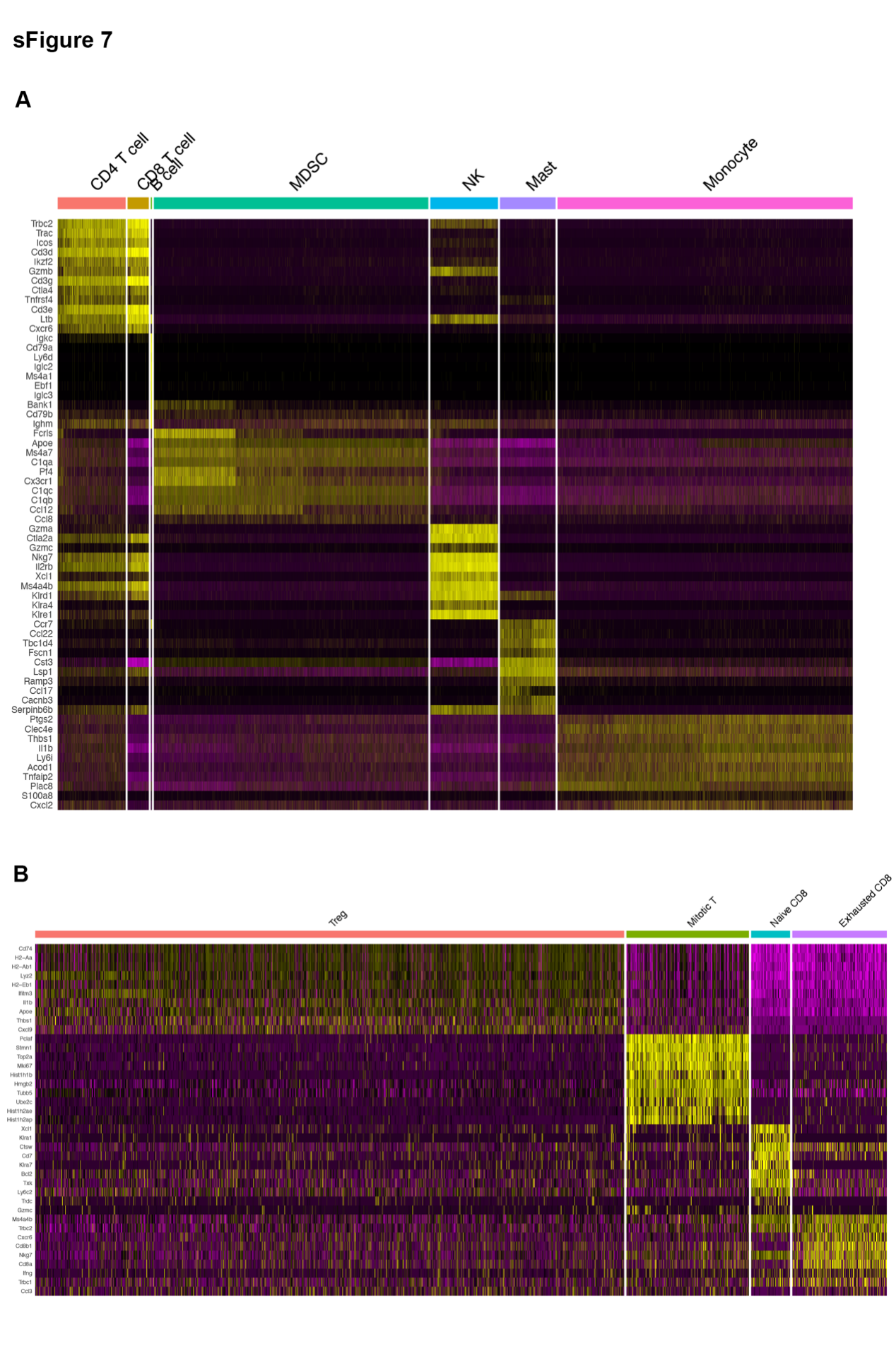


**Supplementary Figure 7. A** and **B,** The heat map shows the different genes in clusters of CD45^+^ cell from KC and KC;*Ubr7^-/-^* tumor.


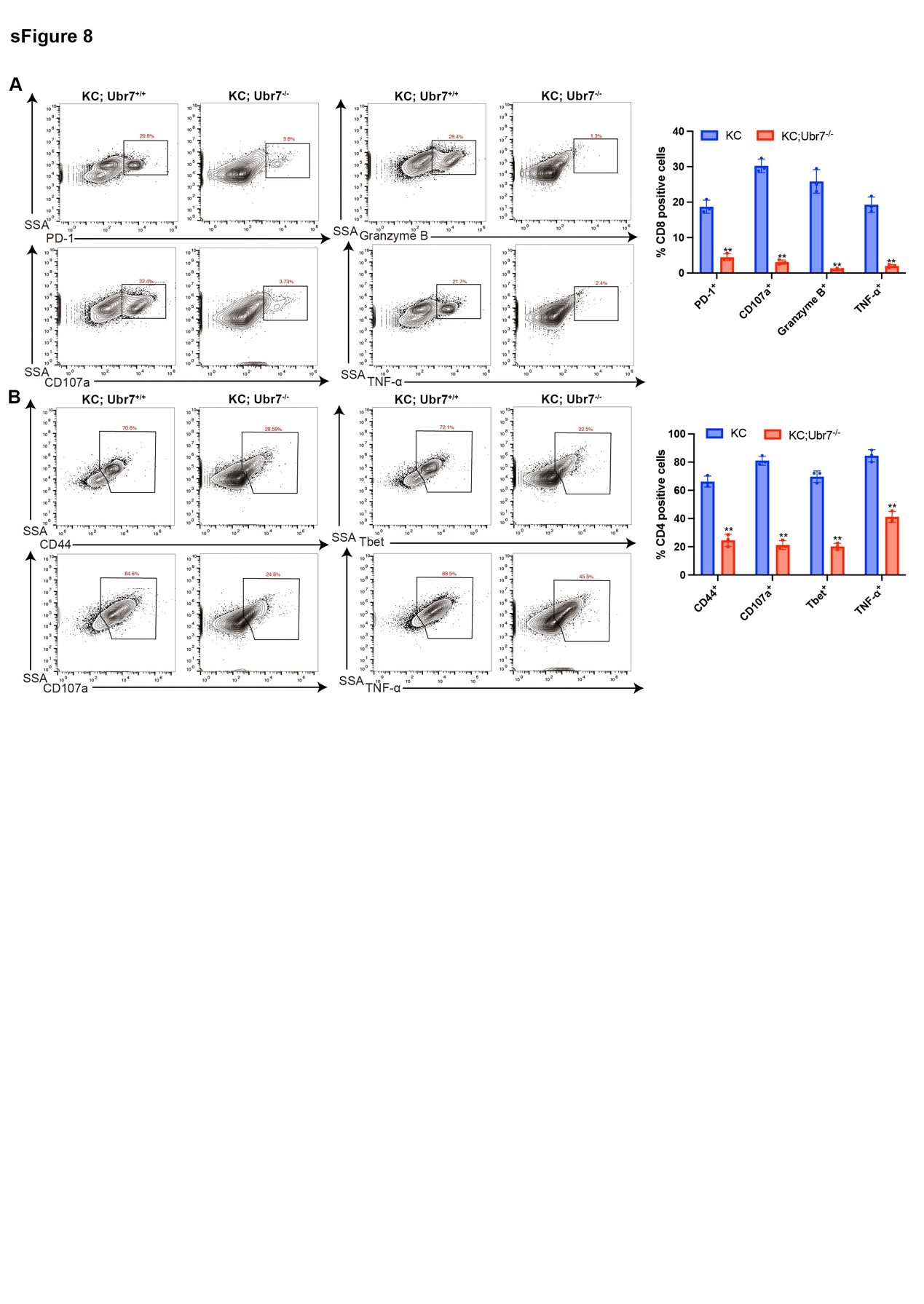


**Supplementary Figure 8. A,** Flow cytometry was used to detect the levels of PD-1, Granzyme B, CD107a and TNF-α secreted by CD8^+^ T cells in tumor tissues form KC and KC;*Ubr7^-/-^* tumor. **B,** Flow cytometry was used to detect the levels of CD44, Tbet, CD107a and TNF-α secreted by CD4^+^ T cells in tumor tissues form KC and KC;*Ubr7^-/-^* tumor. **P < 0.01. Data are presented as mean ± standard error.


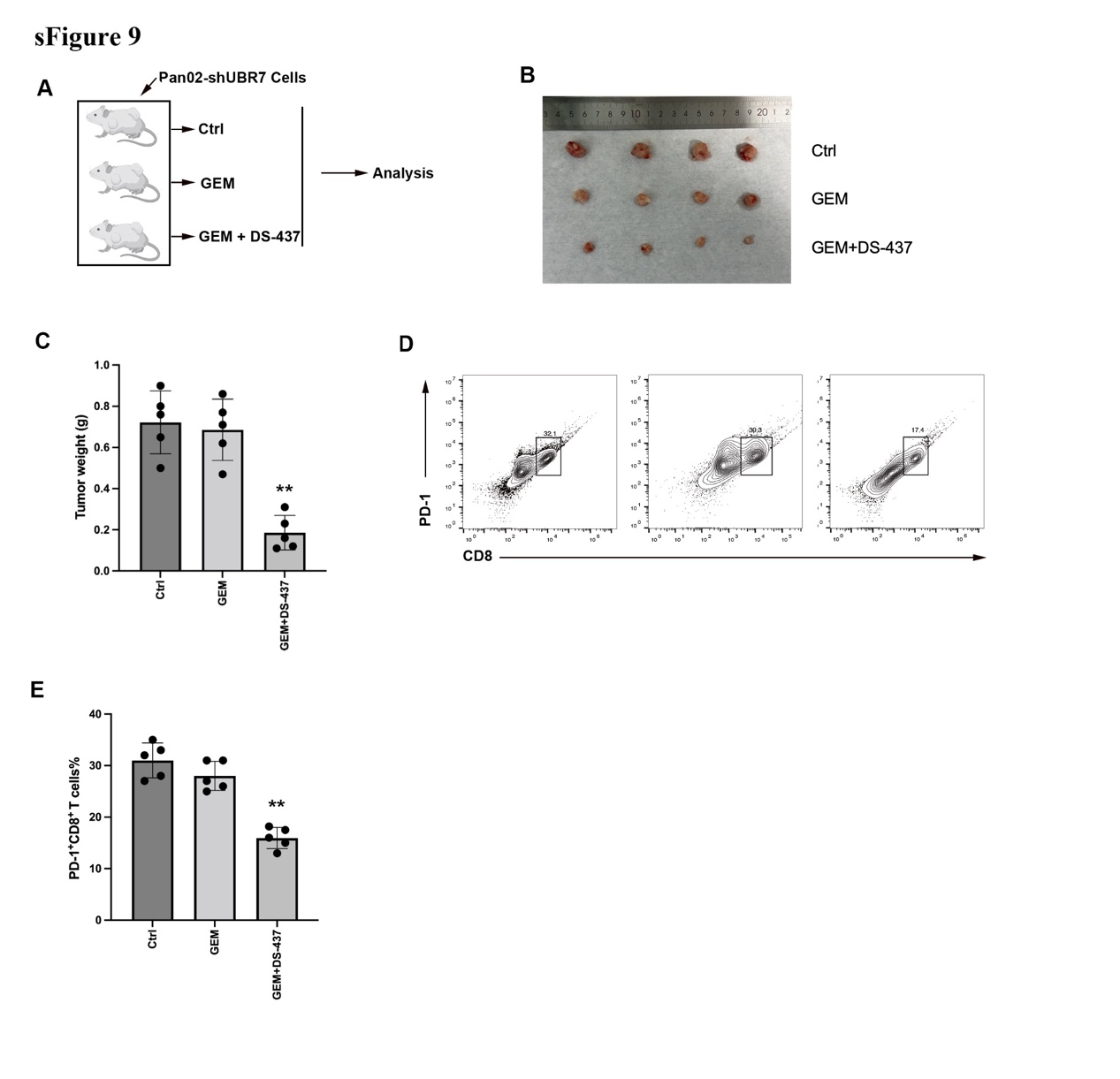


**Supplementary Figure 9. Inhibition of PRMT5 effectively reverses GEM resistance in PDAC cells. A,** Pan02-shUBR7 cells were injected subcutaneously into C57BL/6 mice, and treated with GEM or GEM combined with DS-437 (PRMT5 inhibitor), respectively. **B** and **C,** Subcutaneous tumor morphology (B) and size (C) in mice with designated treatment. **D** and **E,** Flow cytometry analysis (D) and quantification (E) of PD-1^+^CD8^+^ T cells in mice with designated treatments. **p < 0.01, ***p < 0.001. Error bars represent the standard error for three technical replicates.
