# Supplementary material for: The interaction between UBR7 and PRMT5 drives PDAC resistance to gemcitabine by regulating glycolysis and immune microenvironment
Source: Cell Death Dis. 2024 Oct 18;15(10):758. doi: 10.1038/s41419-024-07145-z (PMC11489413; doi:10.1038/s41419-024-07145-z)

Figure 3I

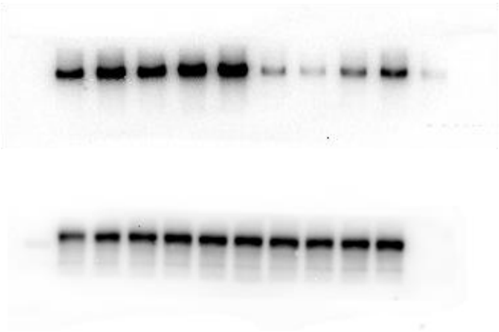

UBR7

Actin

Figure 4D

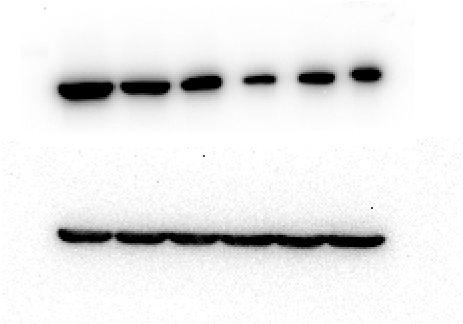

UBR7

Actin

Figure 4F

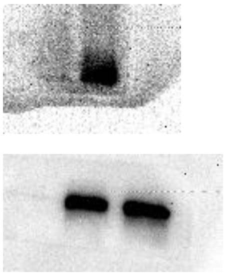

UBR7

Actin

Figure 4I

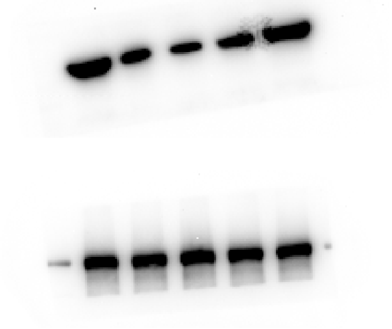

UBR7

Actin

Figure 6B

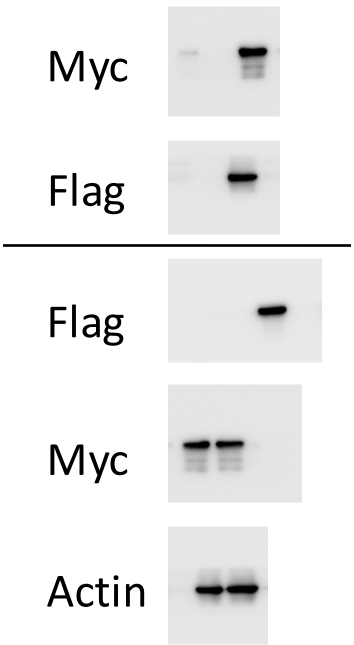

Figure 6C

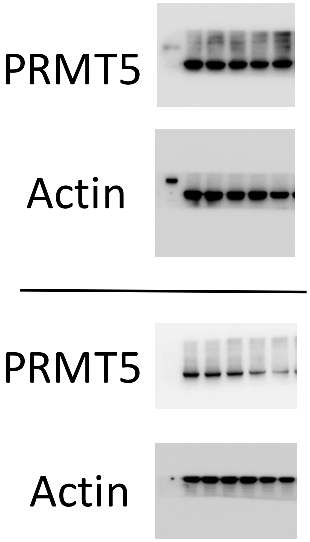

Figure 6E

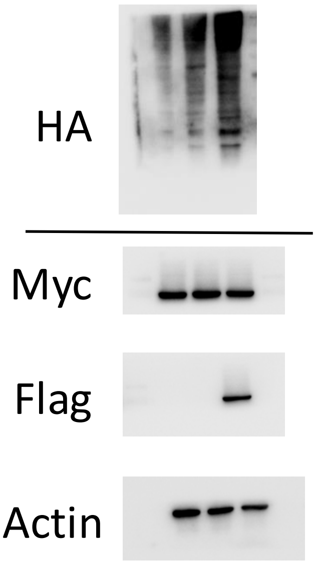

Figure 6F

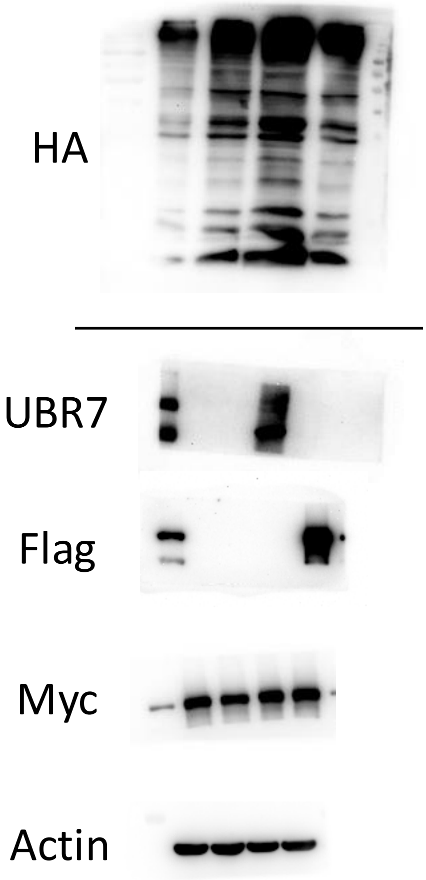

Figure 6G

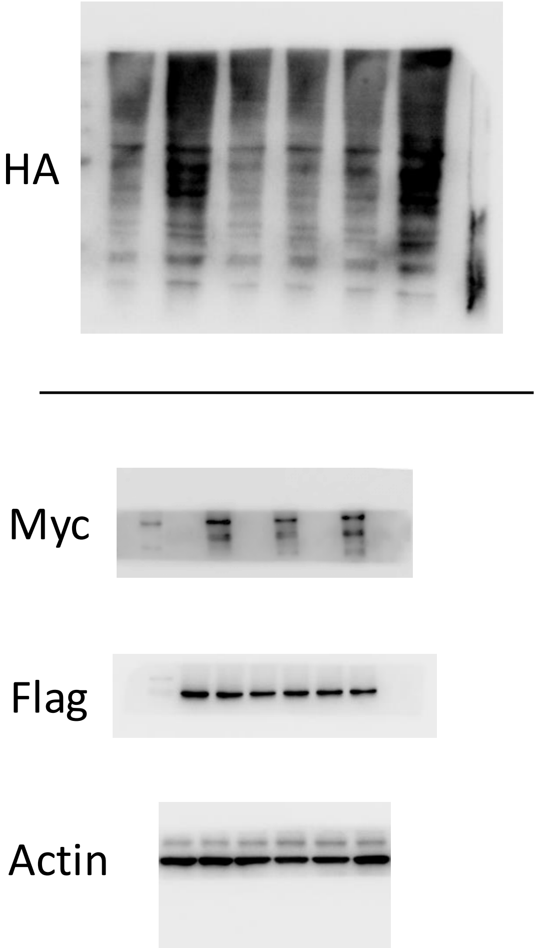

Figure 6H

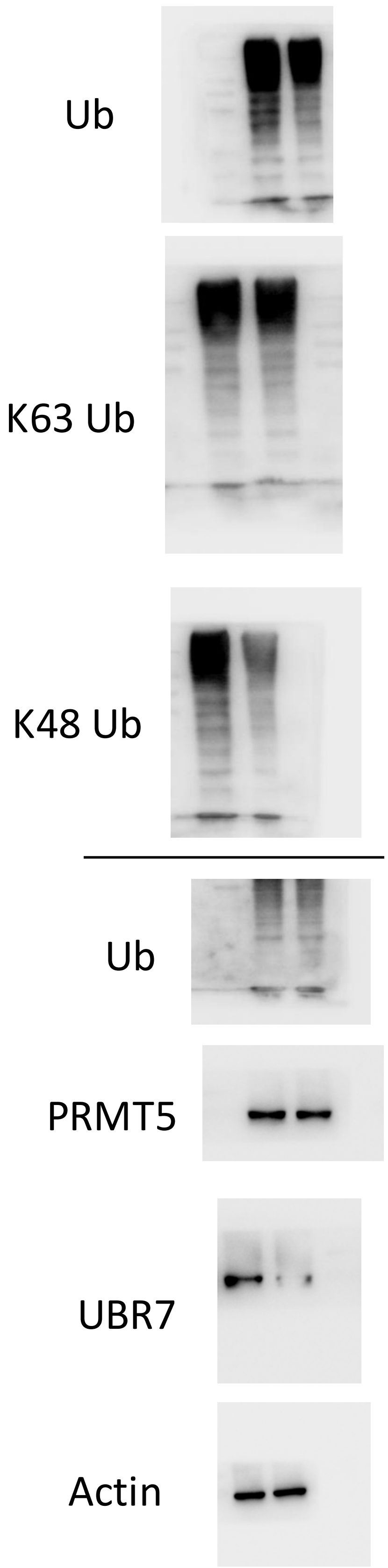

Figure 6I

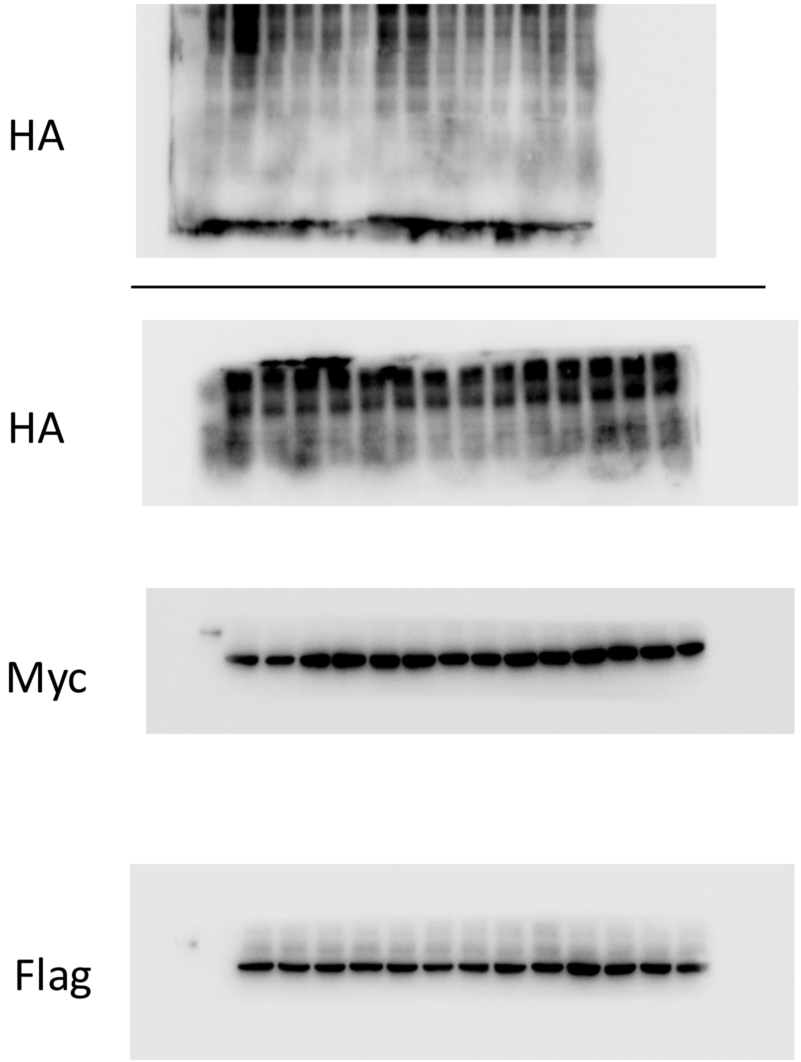

Figure 7C

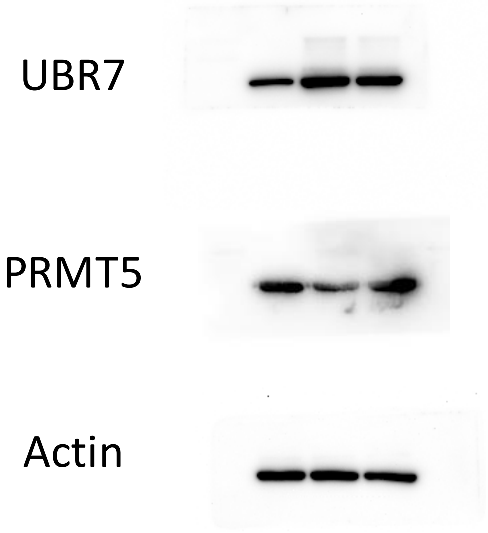

Figure 8J

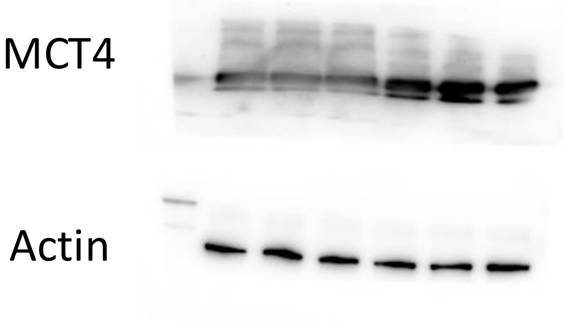

Figure 8K

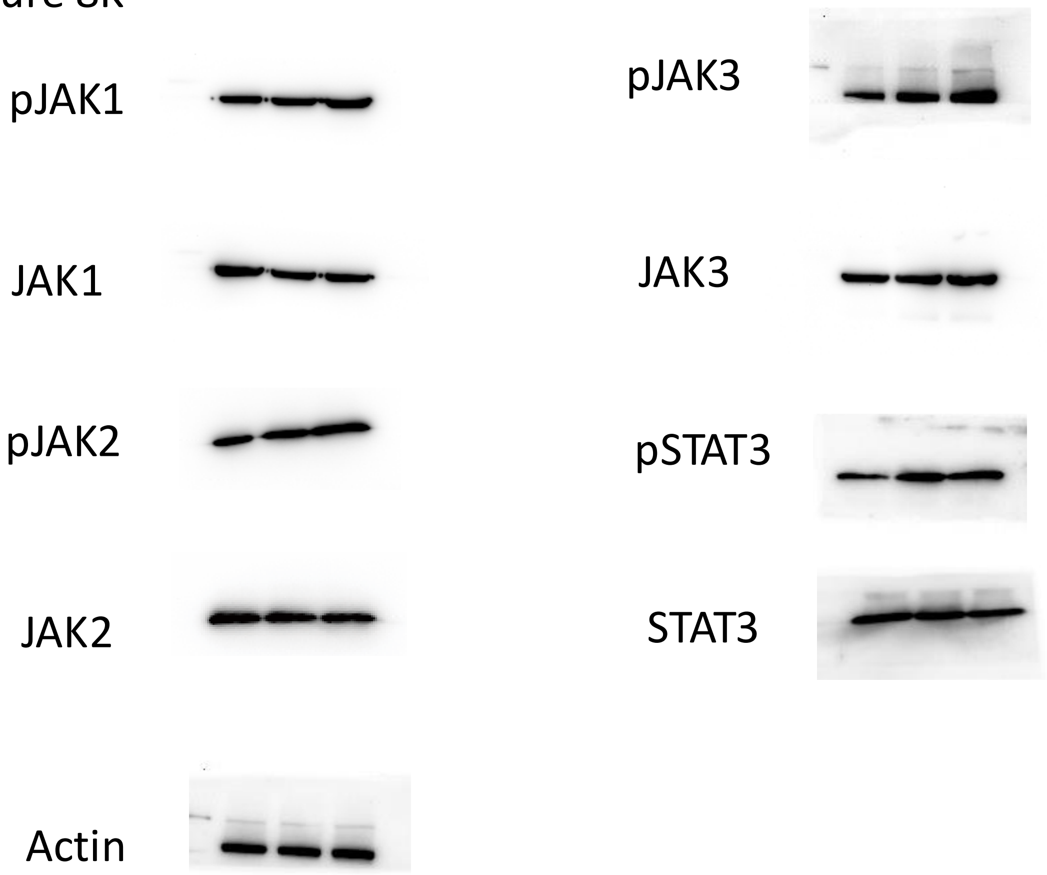

sFigure 1B

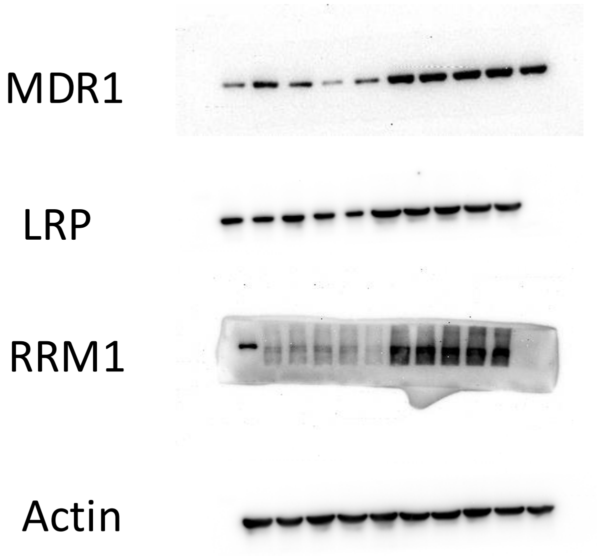

sFigure 4A

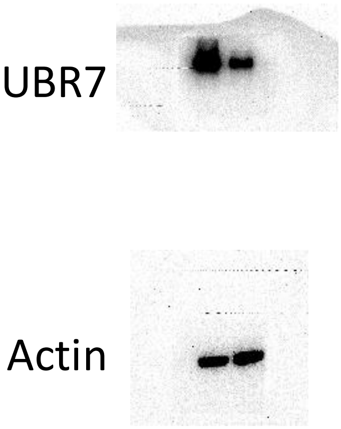

sFigure 5B

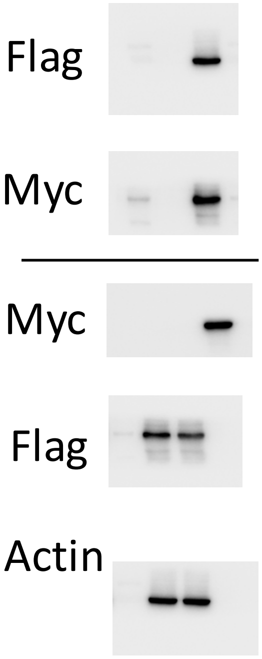

sFigure 5C

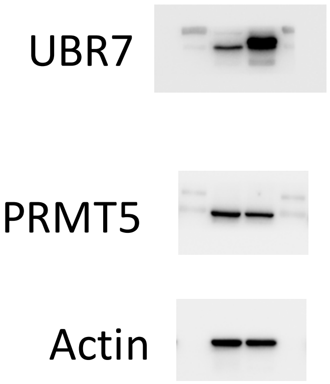

sFigure 5D

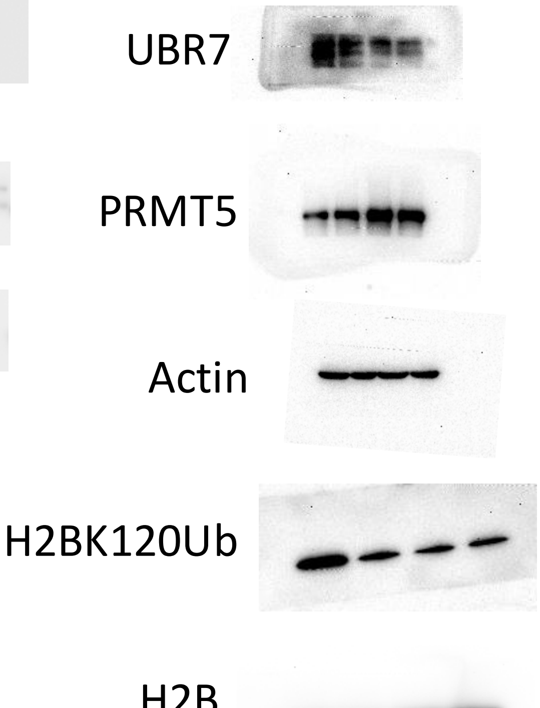

sFigure 5E

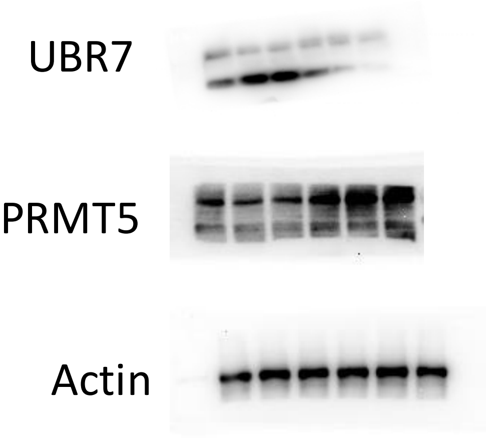

sFigure 5I

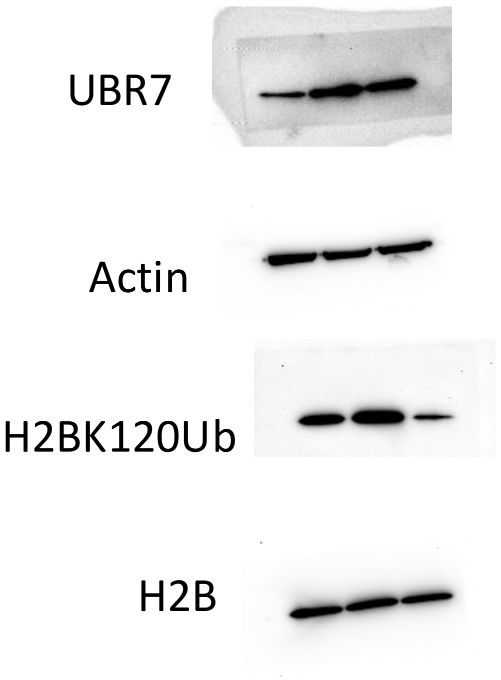

sFigure 6A

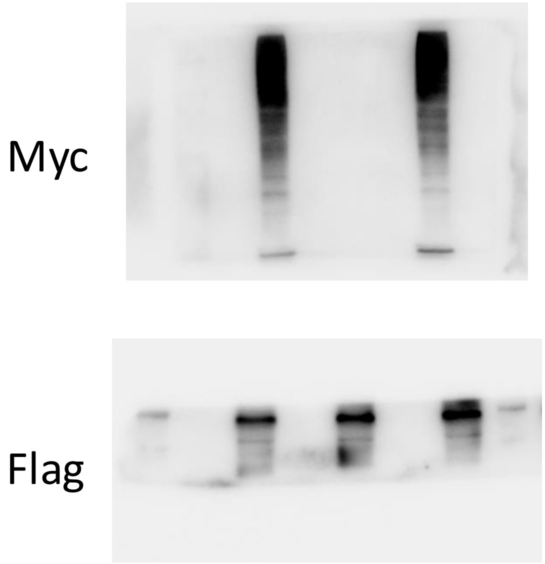

sFigure 6B

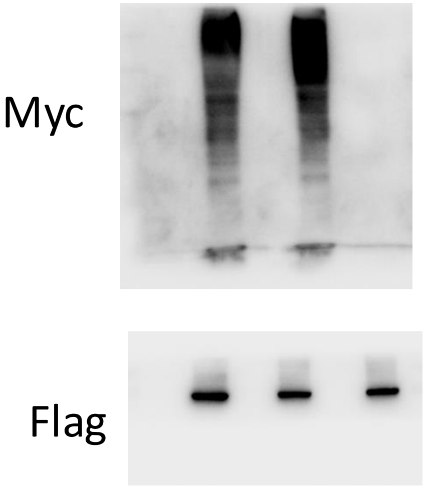

sFigure 6D

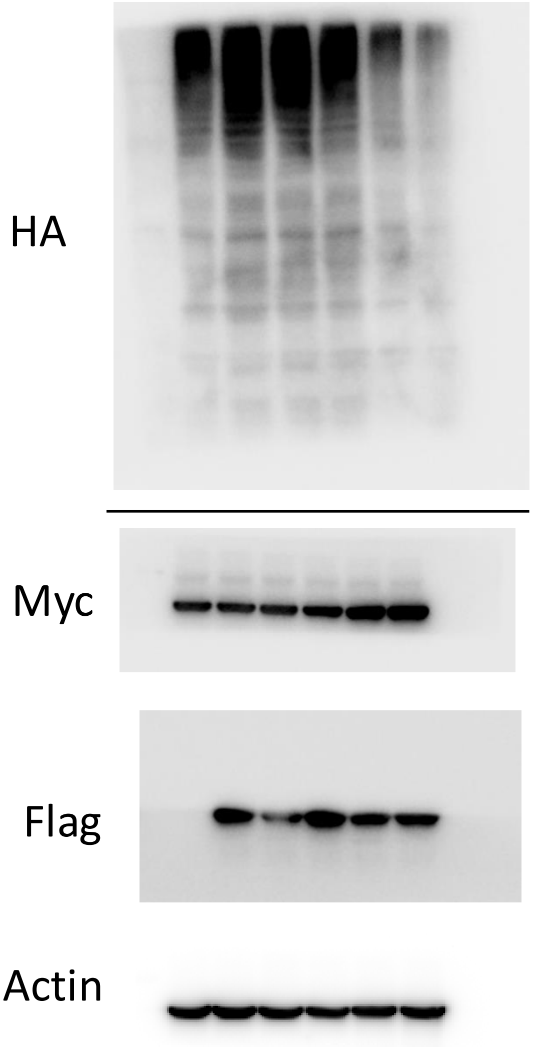

Supplement: Supplementary file 2 — Original Data [file 41419_2024_7145_MOESM2_ESM.pdf]
